# From rhizome diffusion to programmable architecture: a systems framework for controllable spatial expansion in bamboo and other clonal plants

**DOI:** 10.3389/fpls.2026.1932797

**Published:** 2026-09-18

**Authors:** Wentao Huang, Changan Hu, Shuoli Zheng, Jun Lu, Baibing Chen, Xiang Chen, Jinkun Hu, Zeyi Ouyang, Donglv Shu

**Affiliations:** Institute of Flower Research, Hunan Botanical Garden, Changsha, Hunan, China

**Keywords:** biosafety, bud bank dynamics, carbon allocation, clonal plant architecture, evidence appraisal, genome editing, hormonal regulation, rhizome diffusion

## Abstract

**Introduction:**

Rhizome-driven spatial expansion in bamboo and other clonal plants is governed jointly by architectural patterning, belowground bud bank demography, hormonal crosstalk, carbon allocation, environmental plasticity and genomic regulation. This integrative review synthesises those strands and then asks a question the field has not yet answered explicitly: how much of the mechanistic framework now invoked for programmable rhizome control has actually been demonstrated in a woody bamboo?

**Methods:**

Literature was retrieved from Web of Science, Scopus, PubMed and CNKI and appraised along two axes — the biological provenance of each observation (woody bamboo; other rhizomatous or stoloniferous perennial; annual, herbaceous or model system) and its causal strength (perturbation-based, correlative, or conceptual).

**Results:**

The appraisal shows that architectural description, running-versus-clumping contrasts and clonal integration are supported by causal or quantitative evidence in bamboo itself, whereas the auxin -cytokinin -strigolactone module, BRANCHED1/*TB1* repression, *KNOX*-mediated architectural control and the Tre6P -*SnRK1* -*TOR* growth switch are supported in bamboo correlatively or not at all, having been imported from Arabidopsis, rice, potato, poplar and Nicotiana.

**Discussion:**

On that basis we propose a three-tier genome-editing framework ordered by intervention depth, and argue that in bamboo the binding constraint is delivery rather than target choice: because gregarious monocarpic flowering precludes segregation-based transgene removal, ribonucleoprotein editing of cis-regulatory elements in a transformable *Dendrocalamus* or *Bambusa* background is presently the only route to a transgene-free edited plant, and vegetative containment necessarily dominates reproductive containment. We identify five quantities that the current corpus does not report, including lateral extension rate with variance, barrier breach probability as a function of embedment and inspection interval, and the minimum regenerative rhizome fragment size, and convert them into a staged, falsifiable research agenda with explicit go/no-go criteria.

## 1 Introduction

The spatial expansion of rhizomatous systems has been consistently characterized as a functionally integrated process governed by architectural patterning, bud bank dynamics, hormonal regulation, metabolic allocation, and genomic control across bamboo and other clonal plants. Classical architectural analysis demonstrated that rhizomatous branching follows predictable angular and spacer rules. These rules determine substrate exploration efficiency and meristem accumulation. Consequently, they directly influence horizontal expansion distance and ramet density (Bell and Tomlinson, 1980). In woody bamboos, contrasting monopodial (running) and sympodial (clumping) rhizome systems were shown to differ fundamentally in branching mode, physiology, and invasiveness. This confirms that object boundary definition is essential before any control strategy is discussed (Gutiérrez and Rios, 2024). Bud bank ecology has further clarified that regeneration capacity is determined by natality, dormancy proportion, and depletion rates of belowground buds. These factors together define resilience after disturbance and recovery following rhizome cutting (Ott et al., 2019). Hormonal regulation has been demonstrated to modulate rhizome bud activation. High IAA levels and specific IAA/ABA ratios were positively correlated with shoot emergence and year-round bud germination in bamboo. This indicates that apical dominance and dormancy balance constitute quantifiable control indices (Mao et al., 2024). It should be stated at the outset, however, that this bamboo evidence is correlative: hormone concentrations were measured alongside bud phenology, and no perturbation experiment has yet tested causality in a bamboo rhizome. Similar hormonal regulation through ABA-GA interactions promoting rhizome elongation was identified in perennial grasses. This supports cross-species conservation of control mechanisms (Huang et al., 2025). Systems-wide multi-omics comparisons in rhizomatous rice identified rhizome-specific transcription factors, hormone metabolism genes, and metabolite profiles. This reinforces that spatial expansion is under coordinated transcriptional regulation rather than merely structural growth (He et al., 2014). Advances in bamboo genomics have further revealed regulatory networks controlling rhizome development, internode elongation, and stress responses. CRISPR-based genome editing has been proposed as a feasible pathway toward reversible and directional expansion control (Wang et al., 2025a).

Metabolic allocation has also been demonstrated to function as a quantitative driver of expansion scale. Elevated soluble sugars, starch, nitrogen metabolites, and sucrose phosphate synthase activity were associated with stronger rhizome proliferation capacity in Kentucky bluegrass. This links underground biomass allocation with horizontal spread rate (Ran et al., 2023). Non-structural carbohydrate mobilization in bamboo was shown to regulate gene expression associated with cell division and elongation during rapid shoot and rhizome growth (Chen et al., 2025). At the ecological scale, clonal integration was demonstrated to facilitate resource translocation among ramets. This buffers stress and enhances invasiveness, directly affecting boundary stability and directional spread (Liu et al., 2016). Experimental severing of rhizome connections reduced invasive proliferation. This indicates that physiological integration constitutes a manipulable control target (Otfinowski and Kenkel, 2008). Polyploidization has been shown to enhance rhizome biomass allocation and promote a shift toward guerrilla growth forms. This increases expansion distance and invasiveness in *Solidago canadensis*, suggesting that genomic constitution influences expansion strategy (Feng et al., 2022). Comparable spatial strategy plasticity between phalanx and guerrilla architectures was observed in *Phragmites australis* along environmental gradients. This demonstrates that internode length, branch angle, and ramet density respond predictably to soil moisture and salinity drivers (Jiao et al., 2020). In highly invasive geophytes such as *Polygonum cuspidatum* and Sorghum halepense, rhizome fragmentation and extensive carbohydrate-storing rhizomes were identified as primary mechanisms of rapid regional expansion and persistence after disturbance. This highlights the importance of regeneration speed as a control metric (Barney et al., 2006; Paterson et al., 2020). Collectively, rhizome expansion should be quantitatively framed through architectural parameters, bud activation ratios, hormonal balance, metabolic allocation, clonal integration strength, and genomic constitution. This multi-scale synthesis provides a mechanistic foundation for distinguishing limitation control, directional guidance, and potentially reversible genomic-level regulation of bamboo rhizome diffusion.

Two features distinguish the present treatment from earlier syntheses of clonal growth. First, the mechanistic literature assembled above is drawn from taxonomically heterogeneous systems, and its transfer to woody bamboo is frequently assumed rather than argued. We therefore appraise every mechanism explicitly for provenance and causal strength (Section 1.1), report the outcome as a structured matrix (Section 5, **Figure 1**, **Table 1**), and allow that appraisal, rather than narrative plausibility, to determine which interventions are proposed. Second, we translate the resulting picture into an operational framework: a three-tier genome-editing scheme ordered by intervention depth and scored against bamboo’s own transformation, regeneration and generation-time constraints (Section 4.2, **Figure 2**, **Table 2**), and a staged experimental roadmap with explicit go/no-go decision criteria (Section 4.5, **Table 3**). The review therefore aims not to assert that programmable rhizome architecture is achievable, but to specify precisely what would have to be measured, and in what order, before that claim could be made.

**Figure 1:**
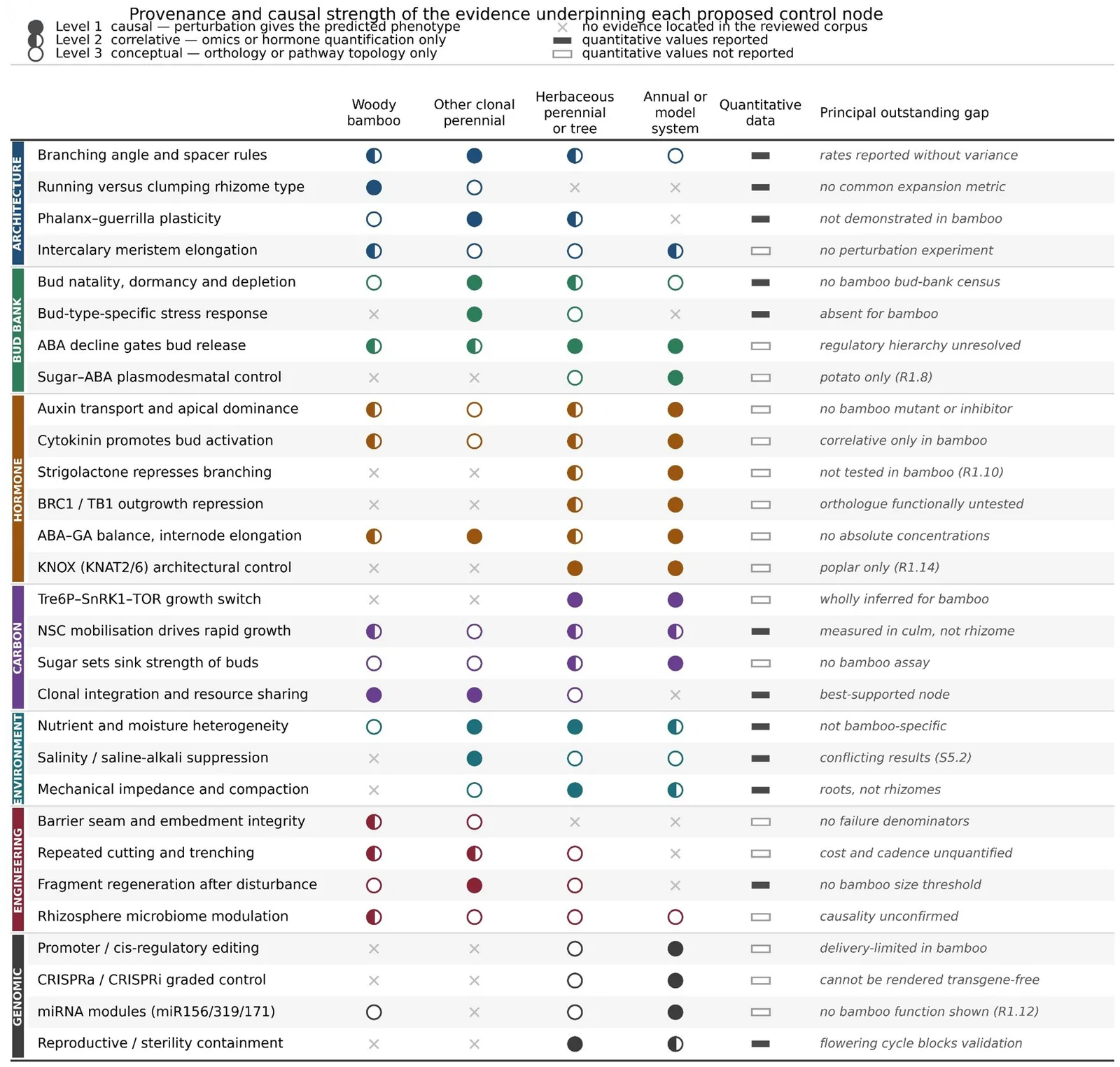
Provenance and causal strength of the evidence underpinning each proposed control node. Rows are the twenty-nine regulatory and management nodes invoked in Sections 2 to 4, grouped by domain. The four columns record the biological system in which the supporting observations were made. Marker fill encodes causal level: filled circle, Level 1 causal evidence in which a genetic or pharmacological perturbation produced the predicted phenotype; half-filled circle, Level 2 correlative evidence from transcriptomic, metabolomic or hormone-quantification studies; open circle, Level 3 conceptual evidence inferred from orthology or pathway topology alone; cross, no evidence located in the reviewed corpus. The adjacent square indicates whether quantitative values were reported. The italic column states the principal outstanding gap for each node. The distribution of filled markers in the first column is the central empirical result of this review. the exact number of nodes per category and the underlying references are given in **Table 1**; the visual matrix above is a schematic summary. In summary, across the twenty-nine nodes scored, two carry Level 1 evidence in Bambusoideae, four carry Level 1 evidence in Tier B systems only, and the remaining nodes are supported by Level 2 correlative or Level 3 conceptual evidence.

**Table 1:** Evidence provenance, causal level and quantification status for the twenty-nine control nodes invoked in this review.

Control node	Domain	Best evidence in woody bamboo (Tier A)	Best evidence in any other system	Quantitative values reported	Principal outstanding gap
Branching angle and spacer rules	Architecture	Level 2	Level 1 in other clonal perennials	Yes	rates reported without variance
Running versus clumping rhizome type	Architecture	Level 1	Level 3 elsewhere	Yes	no common expansion metric across studies
Phalanx-guerrilla plasticity	Architecture	Level 3	Level 1 in Phragmites and other clonal graminoids	Yes	not demonstrated in bamboo; habit may be canalised
Intercalary meristem elongation	Architecture	Level 2	Level 2 in annual grasses	No	no perturbation experiment in bamboo
Bud natality, dormancy and depletion	Bud bank	Level 3	Level 1 in clonal graminoids	Yes	no bamboo bud-bank census
Bud-type-specific stress response	Bud bank	none located	Level 1 in Leymus chinensis	Yes	absent for bamboo
ABA decline gates bud release	Bud bank	Level 2	Level 1 in trees and annual models	No	regulatory hierarchy among ABA, auxin, cytokinin and sugar unresolved
Sugar-ABA plasmodesmatal control	Bud bank	none located	Level 1 in potato	No	Tier C only; not tested in any perennial rhizome
Auxin transport and apical dominance	Hormone	Level 2	Level 1 in annual models	No	no bamboo mutant or transport-inhibitor experiment
Cytokinin promotes bud activation	Hormone	Level 2	Level 1 in rice and Arabidopsis	No	correlative only in bamboo
Strigolactone represses branching	Hormone	none located	Level 1 in Arabidopsis, rice and Nicotiana	No	not tested in bamboo despite being a central premise
*BRC1*/*TB1* outgrowth repression	Hormone	none located	Level 1 in annual models	No	bamboo orthologue annotated but functionally untested
ABA-GA balance and internode elongation	Hormone	Level 2	Level 1 in perennial grasses and cereals	No	no absolute node-resolved concentrations
*KNOX* (*KNAT2/6*) architectural control	Hormone	none located	Level 1 in poplar	No	poplar is a non-clonal tree; bamboo orthologues uncharacterised
*Tre6P-SnRK1-TOR* growth switch	Carbon	none located	Level 1 in Arabidopsis and cereals	No	Tre6P has never been quantified in a bamboo rhizome
NSC mobilisation drives rapid growth	Carbon	Level 2	Level 2 in other perennials	Yes	measured in culm, not in rhizome
Sugar sets sink strength of buds	Carbon	Level 3	Level 1 in annual models	No	no bamboo assay
Clonal integration and resource sharing	Carbon	Level 1	Level 1 in other clonal perennials	Yes	best-supported node in the whole framework
Nutrient and moisture heterogeneity	Environment	Level 3	Level 1 in clonal graminoids	Yes	not bamboo-specific
Salinity and saline-alkali suppression	Environment	none located	Level 1 in Leymus chinensis	Yes	direction of response conflicting between studies (Section 5.2)
Mechanical impedance and compaction	Environment	none located	Level 1 in crop root systems	Yes	concerns roots, not rhizomes; deflection geometry unquantified
Barrier seam and embedment integrity	Engineering	Level 2	Level 2 in other invasive rhizomatous species	No	no failure denominators, so no reliability function
Repeated cutting and trenching	Engineering	Level 2	Level 2 in other clonal invaders	No	required cadence, duration and cost unquantified
Fragment regeneration after disturbance	Engineering	Level 3	Level 1 in *Polygonum cuspidatum* and *Butomus umbellatus*	Yes	no minimum fragment size threshold for any bamboo
Rhizosphere microbiome modulation	Engineering	Level 2	Level 2 elsewhere	No	wholly observational; direction of causality unresolved
Promoter/cis-regulatory editing	Genomic	none located	Level 1 in rice and other crops	No	delivery-limited in bamboo; no rhizome-preferential promoters characterised
CRISPRa/CRISPRi graded control	Genomic	none located	Level 1 in annual models	No	cannot be rendered transgene-free, so a validation tool only
miRNA modules (*miR156/319/171*)	Genomic	Level 3	Level 1 in rice, maize and Arabidopsis	No	no bamboo miRNA shown to alter rhizome architecture
Reproductive and sterility containment	Genomic	none located	Level 1 in woody ornamentals	Yes	multi-decadal flowering cycle blocks pre-release validation

Causal levels are defined in Section 1.1. Of the twenty-nine nodes, two carry Level 1 causal evidence obtained in Bambusoideae. The same scoring is shown graphically in **Figure 1**.

**Figure 2:**
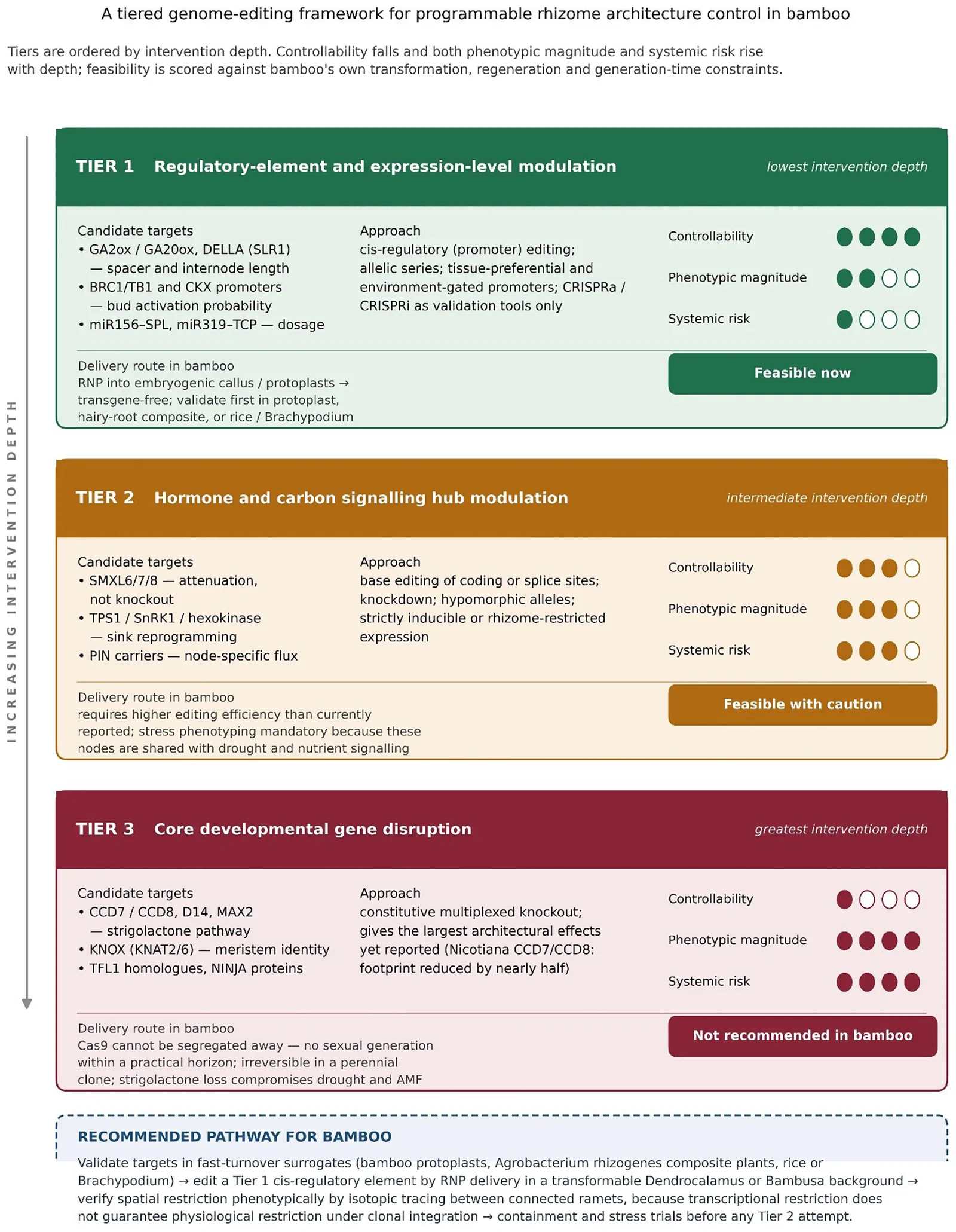
Tiered genome-editing framework for programmable rhizome architecture control in bamboo. Tiers are ordered by intervention depth. Tier 1 comprises cis-regulatory and expression-level modulation of gibberellin metabolism, bud-outgrowth repressors and cytokinin catabolism genes; Tier 2 comprises modulation of hormone and carbon signalling hubs; Tier 3 comprises constitutive disruption of core developmental genes. Filled circles indicate the relative level of controllability, phenotypic magnitude and systemic risk on a four-point ordinal scale derived from the cross-species evidence reviewed in Section 4.2. The feasibility badge and the delivery-route footer of each tier score the tier against bamboo’s own transformation, regeneration and generation-time constraints. The banner summarises the recommended sequence of work, in which spatial restriction is verified physiologically by isotopic tracing between connected ramets rather than by promoter–reporter assay alone. The feasibility assessments shown reflect current technology and bamboo-specific constraints; they are not predictions of successful implementation in any given genetic background.

**Table 2:** Tiered genome-editing options for rhizome architecture control, with bamboo-specific feasibility assessment.

Tier	Intervention class and named candidate targets	Editing approach	Expected architectural effect	Controllability/phenotypic magnitude/systemic risk	Feasibility under bamboo-specific constraints
Tier 1	Regulatory-element and expression-level modulation. *GA2ox/GA20ox* and *DELLA* (*SLR1*) for spacer and internode length; *BRC1*/*TB1* and *CKX* promoters for bud activation probability; *miR156-SPL* and *miR319-TCP* dosage	cis-regulatory (promoter) editing; promoter allelic series; tissue-preferential or environment-gated promoters; CRISPRa and CRISPRi used as validation tools only	Graded shortening of spacers and graded reduction of bud activation, reversible in principle and dose-tunable	Controllability: high. Phenotypic magnitude: low to moderate. Systemic risk: low	Delivery component feasible with current RNP technology; however, the complete Tier 1 pipeline awaits the nomination of rhizome-preferential promoters, which have not yet been characterised in bamboo and are a prerequisite deliverable of Stage 1 (**Table 3**).
Tier 2	Hormone and carbon signalling hub modulation. *SMXL6/7/8* attenuation rather than knockout; *TPS1*, *SnRK1* and hexokinase for sink reprogramming; *PIN* carriers for node-specific auxin flux	Base editing of coding or splice sites; knockdown; hypomorphic alleles; strictly inducible or rhizome-restricted expression	Substantial redistribution of assimilate between expansion, storage and stress maintenance; stronger architectural change than Tier 1	Controllability: moderate. Phenotypic magnitude: moderate to high. Systemic risk: moderate to high	Feasible with caution. Requires higher editing efficiency than currently reported for any bamboo. These nodes are shared with drought and nutrient signalling, so full stress phenotyping is mandatory rather than optional
Tier 3	Core developmental gene disruption. *CCD7/CCD8, D14* and *MAX2* in the strigolactone pathway; *KNOX* genes (*KNAT2/6*); *TFL1* homologues and *NINJA*-domain proteins	Constitutive multiplexed knockout	Largest architectural effects reported in any system (in Nicotiana, *CCD7/CCD8* knockout reduced plant spatial footprint by nearly half)	Controllability: low. Phenotypic magnitude: high. Systemic risk: high	Not recommended in bamboo. Hexaploidy requires inactivation of three homoeologues; the editing construct cannot be segregated away because no sexual generation occurs within a practical horizon; the change is irreversible in a clone that persists for decades; and strigolactone loss compromises drought tolerance and arbuscular mycorrhizal symbiosis

Controllability, phenotypic magnitude and systemic risk are ordinal judgements derived from the cross-species evidence reviewed in Section 4.2; they are not measured quantities. The feasibility column is scored against bamboo’s own transformation, regeneration and generation-time constraints (Section 4.2.1). See also **Figure 2**.

**Table 3:** Staged experimental roadmap for validating controllable rhizome architecture in bamboo, with go/no-go decision criteria.

Stage	Objective	Indicative duration	Components and outputs	Go/no-go decision criterion
1	Baseline quantification and target nomination. Requires no transformation capability and should proceed immediately	1–3 years	(a) Rhizome architectural census in a defined running species: spacer length, branching angle, bud density and annual lateral extension, each with a dispersion estimate, across age classes and site types. (b) Node-resolved quantification of auxin, cytokinin, strigolactone, abscisic acid and gibberellin along intact rhizomes at dormant, activating and elongating nodes. (tests H2) (c) Tissue-resolved transcriptome and small-RNA sequencing of rhizome apex, intercalary meristem, dormant bud and activating bud, with degradome-supported miRNA target validation in rhizome tissue. (d) Retrospective compilation of barrier installation and failure records to yield a breach-probability function	Proceed if at least one promoter shows a rhizome-preferential expression ratio sufficient for use, and hormone gradients between dormant and activating nodes are resolvable above measurement noise. If no gradient is detectable, the hormonal gating model is unsupported in bamboo and the programme should redirect toward carbon-supply and meristem-competence explanations
2	Causal validation in surrogate and pharmacological systems, before any commitment to bamboo transformation	2–3 years	(a) Transient expression and CRISPRi assays for candidate genes in bamboo protoplasts. (b) Agrobacterium rhizogenes composite plants where the species permits. (c) Orthologue editing in rice or Brachypodium where the orthology relationship is unambiguous. (d) Pharmacological perturbation of intact bamboo rhizomes with gibberellin, gibberellin biosynthesis inhibitors, cytokinin and synthetic strigolactone analogues, scoring internode length and node number separately (tests H1 and H3)	Proceed only for targets whose pharmacological perturbation produces the predicted directional change in bud activation or internode elongation in bamboo itself. A target that succeeds in surrogates but fails this test is not rate-limiting in bamboo and should not be carried forward
3	Editing and containment-relevant phenotyping. First stage requiring transformation capability(tests H5)	3–5 years	(a) Ribonucleoprotein delivery of a validated cis-regulatory edit into embryogenic callus of a transformable *Dendrocalamus* or *Bambusa* background. (b) Regeneration with non-edited tissue-culture regenerants as the control class. (c) Molecular confirmation of the edit and of the absence of integrated DNA. (d) Containerised and rhizotron phenotyping over at least two growth cycles, scoring spacer length, bud activation, minimum regenerative fragment size, seasonal fragment regeneration probability and lateral extension rate. (e) Verification of spatial restriction by carbon and nitrogen isotopic tracing between connected edited and unedited ramets (tests H4)	Proceed if lateral extension is reduced with statistical support and no detectable penalty is observed in drought and cold survival, photosynthetic performance, or culm mechanical properties. A phenotype that reduces expansion only by reducing overall vigour does not satisfy the criterion
4	Confined field evaluation, trade-off quantification and transfer to a monopodial background	5–10 years	(a) Multi-year barriered field trials with rhizome-directed monitoring under defined disturbance regimes (tests H6). (b) Quantification of the expansion-versus-yield exchange rate across a water and nutrient gradient in edited and unedited material, closing Gap 5. (c) Rhizosphere community and arbuscular mycorrhizal colonisation as non-target endpoints. (d) Transfer of the validated cis-regulatory edit into a monopodial *Phyllostachys* background as transformation capability in that genus improves	Deployment decisions should be staged from containerised through confined to semi-open cultivation, with rhizome-directed monitoring at each step. Regulatory engagement should begin at Stage 3 rather than Stage 4, since dossier requirements for a clonally propagated, transgene-free edited perennial are unsettled in most jurisdictions and will shape the phenotyping design

Hypothesis labels H1–H6 refer to Section 6; H2 is tested in Stage 1, H5 is the core premise of Stage 3. Durations are indicative and reflect bamboo’s growth rhythm rather than a funding cycle.

### 1.1 Scope, literature search, and evidence appraisal strategy

This article is an integrative mechanistic review and is not presented as a systematic review in the PRISMA sense. That designation is deliberate. The evidence relevant to rhizome expansion control spans plant architecture, bud bank demography, hormone physiology, carbon signaling, soil engineering, microbial ecology and genome editing; the outcome variables used in these domains (branching angle in degrees, bud natality per unit rhizome length, hormone concentration, containment failure incidence, editing efficiency) are not commensurable, the great majority of the primary studies share no common control treatment, and many of the propositions synthesized here are mechanistic hypotheses rather than estimable effects. The quantitative pooling that justifies PRISMA-compliant methodology is therefore not achievable, and imposing its apparatus would create a misleading impression of statistical rigor. What can and should be done, and what we do here, is to report the retrieval and appraisal procedure explicitly so that coverage and selection bias can be judged by the reader.

Literature was retrieved from Web of Science Core Collection, Scopus, PubMed and CNKI, supplemented by backward and forward citation tracking from key architectural and bud bank papers and by hand-searching the bamboo-specific literature. Search strings combined (i) an organ or growth-form term (rhizome OR stolon OR clonal OR ramet OR 竹鞭), (ii) a process term (expansion OR spread OR invasion OR bud bank OR dormancy OR branching OR internode elongation OR containment), and (iii) where appropriate a mechanism term (auxin OR cytokinin OR strigolactone OR abscisic acid OR gibberellin OR trehalose-6-phosphate OR *SnRK1* OR *TOR* OR non-structural carbohydrate OR CRISPR OR genome editing OR root barrier OR rhizosphere microbiome). Records were retained if they reported original data, a mechanistic model, or a systematic synthesis relevant to horizontal belowground expansion or to its deliberate modification, and were excluded if they addressed aboveground architecture only with no belowground or bud-bank component, if they were purely taxonomic or floristic, or if no methodological detail sufficient for provenance scoring could be recovered. Foundational architectural and meristem literature was retained irrespective of publication date, since the geometric rules of rhizome branching and the anatomy of intercalary meristems are described in older primary sources that have not been superseded. Screening and appraisal were performed independently by two authors and disagreements resolved by discussion with a third.

Every mechanism carried forward was then appraised on two orthogonal axes. The first axis is provenance, which records the biological system in which the observation was actually made: Tier A denotes a woody bamboo (Bambusoideae); Tier B denotes another rhizomatous or stoloniferous perennial, such as Phragmites, Leymus, Solidago, Poa or a rhizomatous Oryza; and Tier C denotes an annual, herbaceous or model system, including Arabidopsis, rice, potato, tomato, Nicotiana and non-clonal woody trees, from which the mechanism is transferred by orthology or analogy. The second axis is causal strength: Level 1 denotes causal evidence, in which a genetic or pharmacological perturbation produced the predicted architectural phenotype; Level 2 denotes correlative evidence, in which transcriptomic, metabolomic or hormone-quantification data covary with the phenotype but no perturbation was performed; and Level 3 denotes conceptual inference drawn from pathway topology or sequence orthology alone. A separate binary flag records whether the source reported quantitative values with a measure of dispersion, as distinct from a qualitative direction of effect.

These descriptors are used throughout Sections 2 to 4, where each principal claim is accompanied by an explicit statement of the system in which it was demonstrated, and they are consolidated in Section 5, where the complete appraisal is presented as a matrix (**Figure 1**) and in tabular form (**Table 1**). Readers who wish to know the evidential status of any single control node before reading the mechanistic sections may consult those two items first. We note in advance the principal result of the appraisal, because it conditions everything that follows: of the twenty-nine control nodes scored, only two carry Level 1 causal evidence obtained in a woody bamboo, and the hormonal module most often invoked as the lever for architectural engineering carries none.

## 2 Molecular and physiological mechanisms

### 2.1 Underground bud bank function and regulation

The underground expansion driven by stolons or rhizomatous axes has been consistently interpreted as a systems-level coupling among resource status, hormonal gradients, meristem competence, and environmental perception. The belowground bud bank functions as a dynamic reservoir of regenerative meristems. Bud banks operate as demographic storage organs whose natality, dormancy, protection, and depletion jointly determine regenerative capacity. They also determine post-disturbance expansion potential. This constrains or enables clonal spread at the population scale (Ott et al., 2019). This demographic framework was developed in North American grassland forbs and graminoids (provenance Tier B) and has not been parameterized for any woody bamboo; no published bud bank census reports natality, dormancy fraction and depletion rate for a *Phyllostachys* or *Dendrocalamus* rhizome system, which is why bud bank density cannot at present be used as a quantitative predictor of bamboo spread rate. Empirical synthesis across geophytes further indicates that bud bank density is generally resilient but selectively plastic under perturbations such as drought or habitat-specific stress. Rhizome and tiller buds respond divergently, suggesting that bud-type identity modulates expansion capacity (Fang et al., 2024). In perennial woody systems, dormancy transitions have been shown to require coordinated shifts in transport processes, non-structural carbohydrate dynamics, hormone signaling, and epigenetic regulation. This forms an integrated seasonal regulatory framework that defines meristem competence thresholds (Fadón et al., 2020). Molecular analyses across tree species reveal that ABA, gibberellins, cytokinins, and auxin collectively regulate endodormancy induction and release. ABA acts as a central repressive signal whose decline is necessary for growth reactivation (Pan et al., 2021; Sabir et al., 2025; Ding et al., 2024). This body of work derives from temperate deciduous trees and is Tier C with respect to bamboo; its transfer assumes that a subterranean bamboo rhizome bud and an aerial flower bud of Prunus share dormancy control logic, an assumption that is plausible but untested. Photoperiodic and temperature cues are transduced through CO/FT modules, circadian networks, and dormancy-associated MADS-box factors. This embeds environmental sensing into meristem fate decisions (Cooke et al., 2012; Zhao et al., 2025). Structural and proteomic studies in conifers demonstrate that plastid organization, starch turnover, and plasmodesmatal status shift markedly between endo- and ecodormancy. This implies that metabolic reprogramming precedes visible bud activation (Guzicka et al., 2018) (**Table 4**).

**Table 4:** Key biological mechanisms regulating rhizome expansion and bud bank dynamics.

Regulatory domain	Key factors/Processes	Specific findings/Observations	Plant system/Model	References	Provenance tier	Causal level
Architectural Patterns	Branching and Internode Rules	Branching angles and internode length determine substrate exploration efficiency and ramet density.	General Rhizomatous Plants	(Bell and Tomlinson, 1980)	B	2
	Rhizome System Types	Fundamental differences in branching mode and invasiveness exist between monopodial (running) and sympodial (clumping) types.	Woody Bamboos	(Gutiérrez and Rios, 2024)	A	1
	Structural Plasticity	Internode length, branching angle, and ramet density vary predictably along environmental gradients (e.g., salinity).	Reed (*Phragmites australis*)	(Jiao et al., 2020)	B	2
Belowground Bud Bank Dynamics	Bud Bank Ecology	Regeneration capacity is determined by bud natality, dormancy, and mortality rates, forming the basis of resilience.	General	(Ott et al., 2019)	B	2
Hormonal Regulation	IAA/ABA Balance	High IAA levels and specific IAA/ABA ratios are positively correlated with year-round bud germination in bamboo.	Bamboo	(Mao et al., 2024)	A	2
	ABA-GA Interactions	ABA-GA interactions promote rhizome elongation in perennial grasses.	Perennial Grasses	(Huang et al., 2025)	B	2
Metabolism and Resource Allocation	C/N Metabolites	Elevated levels of soluble sugars, starch, and nitrogen metabolites correlate with stronger rhizome proliferation capacity.	Kentucky Bluegrass	(Ran et al., 2023)	B	2
	Non-Structural Carbohydrates (NSC)	NSC mobilization regulates genes associated with cell division and elongation driving rapid growth.	Bamboo	(Chen et al., 2025)	A	2
Genomics and Ecological Integration	Multi-Omics Analysis	Rhizome-specific transcription factors and hormone metabolism genes have been identified, demonstrating transcriptional regulation of expansion.	Rhizomatous Rice	(He et al., 2014)	B	2
	Clonal Integration	Clonal integration enhances stress buffering and invasiveness through resource translocation; severing connections reduces invasiveness.	General; Smooth Brome	(Liu et al., 2016; Otfinowski and Kenkel, 2008)	B	1
	Polyploidization	Polyploidization enhances rhizome biomass allocation and promotes guerrilla growth forms, increasing expansion distance.	Canada Goldenrod	(Feng et al., 2022)	B	2
	Genomic Advances	Regulatory networks controlling rhizome development and internode elongation have been revealed, providing targets for gene editing.	Bamboo	(Wang et al., 2025a)	A	3

Provenance tier: A = woody bamboo (Bambusoideae); B = other rhizomatous or stoloniferous perennial; C = annual, herbaceous model system, or non-clonal woody tree. Causal level: 1 = causal, a genetic or pharmacological perturbation produced the predicted phenotype; 2 = correlative, based on omics or hormone quantification; 3 = conceptual, inferred from orthology or pathway topology alone. Definitions are given in Section 1.1.

Convergent evidence from herbaceous perennials and clonal grasses indicates that carbohydrate availability and sugar signaling act not merely as energetic substrates but as regulatory signals that define bud activation thresholds. In rhizomatous bamboo, seasonal bud germination has been associated with increased IAA and zeatin levels, decreased ABA, and upregulation of carbohydrate metabolism genes. This indicates that hormone-carbon coordination drives year-round bud competence (Li et al., 2022). This is the strongest bamboo-derived evidence available for the bud bank node, and it remains Level 2: hormone titers and transcript abundances were measured across a phenological series, but neither hormone application nor genetic perturbation was used to test whether the association is causal. Similar metabolic-hormonal integration has been observed in tea buds. Soluble sugar accumulation and moderate oxidative cues precede sprouting. This is followed by cytokinin-centered activation and rapid carbohydrate consumption (Tang et al., 2023). Mechanistic work in potato demonstrates that chilling-induced sugar accumulation correlates more strongly with dormancy release than chilling units per se. Sugar signaling antagonizes ABA-mediated plasmodesmatal closure, directly modulating symplastic connectivity of dormant meristems (Danieli et al., 2023). The sugar–ABA–plasmodesmata mechanism is, however, a Tier C result obtained in a potato tuber bud, an organ whose symplastic architecture and dormancy duration differ substantially from those of a bamboo rhizome bud; we invoke it here as a candidate mechanism and not as an established feature of bamboo bud release, and its transfer should be tested directly by carboxyfluorescein diacetate movement assays in rhizome nodes before it is used to justify any intervention. Transcriptomic analysis of underground renewal buds in peony further confirms coordinated shifts in ABA/IAA balance, dehydrin expression, and stress-responsive pathways during dormancy transition (Zhang et al., 2015a). This peony dataset is likewise correlative and derives from a herbaceous perennial with determinate renewal buds rather than an indeterminate running rhizome; the shared ABA/IAA signature is therefore evidence of a conserved hormonal correlate of dormancy transition, not evidence that the same balance sets spacer length or lateral extension rate. Light signaling has also been implicated as an integrative regulator of bud outgrowth through interactions with auxin transport and strigolactone pathways. This links canopy cues to subterranean meristem activation (Leduc et al., 2014). Developmental-genetic evidence from Arabis alpina shows that maintenance of a dormant bud zone depends on auxin-mediated apical dominance and BRANCHED1-dependent repression. This demonstrates that bud bank size and activation probability are developmentally programmed yet environmentally modulated (Vayssières et al., 2019). Collectively, these studies suggest that bud bank capacity is not static but emerges from the balance between meristem production, dormancy maintenance, and activation thresholds. These thresholds are defined by carbon status, ABA decline, cytokinin/auxin redistribution, oxidative signaling, and symplastic reconnection. Thus, underground expansion rate is ultimately governed by the systemic integration of resource sensing, hormonal crosstalk, and environmental cue transduction at the level of meristematic competence. The qualification that should accompany this synthesis is that its bamboo-specific component consists of a single correlative dataset; the integrative model is assembled from Tier B and Tier C systems and is best regarded as a hypothesis about bamboo rather than a description of it (see **Figure 1**, rows 5–8) (**Figure 3**).

**Figure 3:**
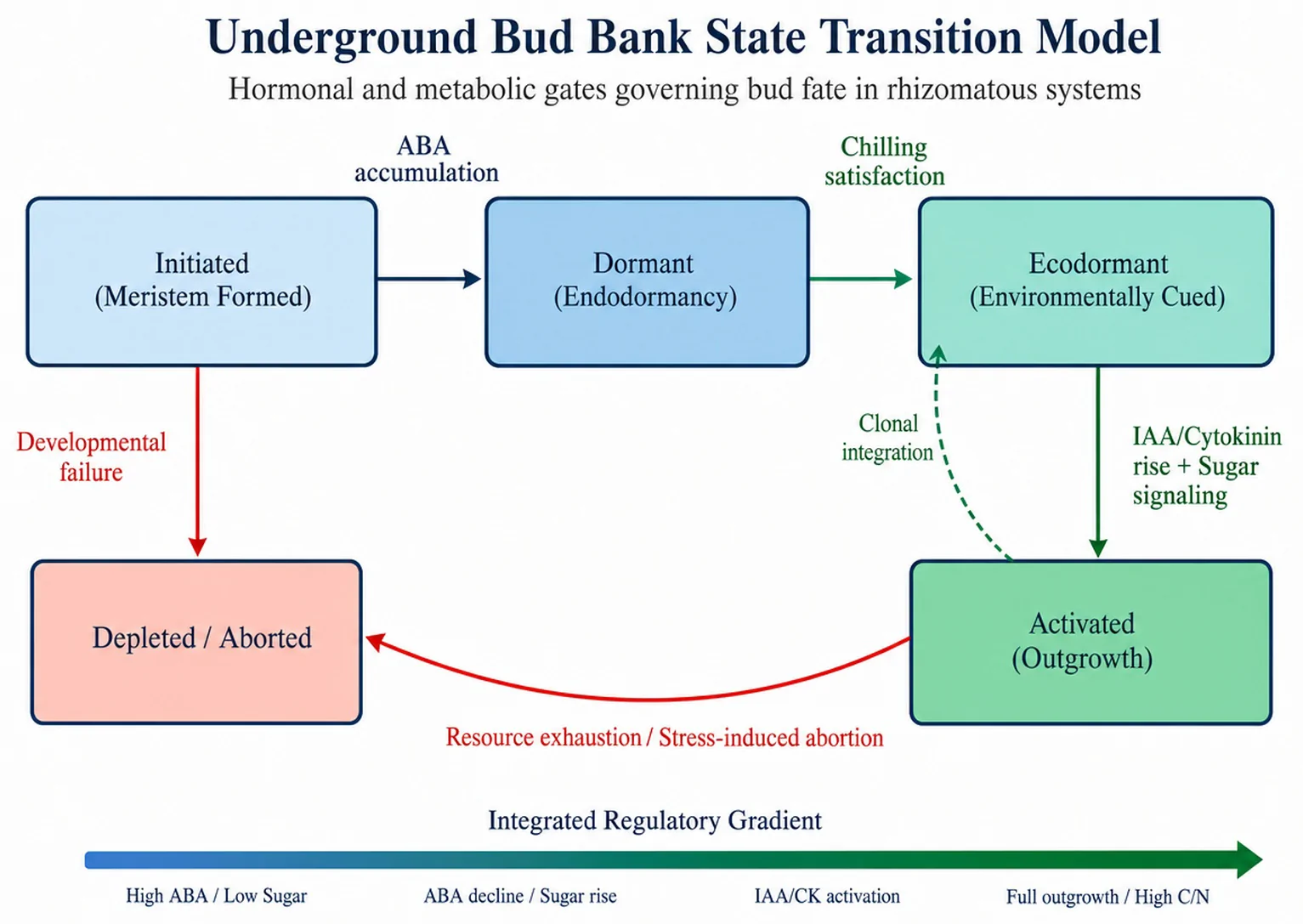
Schematic model of underground bud bank state transitions in rhizomatous plants. Bud fate is determined by the balance among abscisic acid (ABA) accumulation, chilling satisfaction, auxin/cytokinin ratios, and sugar signaling (Tre6P-*SnRK1*). The gradient bar illustrates the integrated hormonal and metabolic regulation from dormancy to activation. Dashed arrow indicates clonal integration feedback from activated ramets. The states and transitions depicted are supported at Level 2 or below in Bambusoideae; the model is presented as an organising hypothesis for bamboo and as a summary of demonstrated behaviour in the Tier B and Tier C systems from which it is drawn.

### 2.2 Hormonal regulation network

The architectural balance between growth promotion and suppression in plants has been consistently interpreted as an emergent property of an integrated hormonal network rather than the dominance of any single phytohormone. Auxin-mediated polar transport establishes local concentration maxima at nodes and stem apices. These maxima condition axillary bud competence and internode elongation through tightly coordinated crosstalk with cytokinins and strigolactones (Mazzoni-Putman et al., 2021; Semerádová et al., 2020). Auxin exported from the shoot apex indirectly suppresses bud outgrowth by modulating cytokinin biosynthesis in the stem and enhancing strigolactone signaling. This reinforces apical dominance. Cytokinins act antagonistically to promote bud activation. This regulatory module is conserved from Arabidopsis to cereals and woody perennials (Schneider et al., 2019; Leduc et al., 2014; Cheng et al., 2013). Strigolactones have been characterized as central repressors of lateral branching and coordinators of shoot and root system architecture. They act through *MAX2*-dependent signaling and interact extensively with auxin transport machinery. This fine-tunes node-specific hormone gradients that determine whether a bud remains dormant or elongates (Rameau et al., 2019; Rehman et al., 2021; Yang et al., 2019; Pandey et al., 2016). Because this module is the principal lever proposed later in this review, its evidential status must be stated without hedging. Every causal demonstration cited above — biosynthesis and perception mutants, hormone application, and reporter analysis — was obtained in Arabidopsis, pea, rice or petunia, all Tier C systems. No causal demonstration that the auxin–cytokinin–strigolactone module gates bud outgrowth in a woody bamboo rhizome currently exists: no bamboo strigolactone biosynthesis or perception mutant has been reported, no strigolactone or synthetic analogue application experiment on bamboo rhizome buds has been published, and the bamboo *BRC1/TB1* orthologue has been identified by sequence and expression but not tested functionally. This is the single most consequential gap identified by the present review, because every downstream editing strategy that targets this module inherits it. In grasses and bamboo, gibberellin-mediated internode elongation has been demonstrated to depend on interactions with auxin and other hormonal pathways. This integrates *DELLA*-dependent growth control with environmental and developmental signals. Internodal extension is hormonally gated at both biosynthetic and signaling levels rather than being a simple GA output (Castro-Camba et al., 2022; Dai et al., 2025). Across dicots and monocots, root and subterranean architecture has likewise been interpreted as a product of overlapping auxin, cytokinin, gibberellin, abscisic acid, ethylene, and strigolactone gene networks. Perturbations in hormonal balance lead to predictable shifts in branching density and elongation patterns (De Smet et al., 2015). A partial exception to the foregoing caution should be recorded: the gibberellin–*DELLA* axis is the one hormonal node for which bamboo-derived evidence approaches Level 1, since GA biosynthesis and signaling genes have been manipulated in bamboo protoplast and heterologous systems and GA application alters internode length in bamboo culms. This asymmetry is the reason the tiered framework proposed in Section 4.2 places GA-metabolism genes, rather than strigolactone pathway genes, in its most immediately actionable tier.

Under drought, salinity, and low-temperature stress, a systemic reprogramming of this growth-suppression equilibrium has been reported. Abscisic acid and ethylene signaling are elevated. Growth-promoting hormones are modulated to prioritize survival over expansion. Hormonal rebalancing alters endogenous levels of auxin, cytokinins, gibberellins, and strigolactones. This reshapes both shoot branching and root exploratory strategies (Waadt et al., 2022; Llanes et al., 2016; Sabagh et al., 2021). ABA-centered stress signaling has been proposed to converge with redox metabolism and strigolactone pathways. This redirects carbon allocation and suppresses lateral outgrowth while enhancing stress-adaptive root traits. This reveals a mechanistic bridge between architectural plasticity and stress resilience (Jardim-Messeder et al., 2025; Yang et al., 2019). In cereals and other crops, auxin-dependent developmental programs have been shown to be reconfigured under abiotic stress. Altered transport and signaling dynamics influence both tillering and root system plasticity. Local hormone gradients at nodes and meristems are stress-sensitive regulatory hubs (Voytenko and Kosakivska, 2025; Mazzoni-Putman et al., 2021). Collectively, comparative evidence across model plants, grasses, legumes, and woody species supports the conclusion that anti-spread or architectural containment in horticultural contexts is best understood not as reduction of a single hormone, but as modulation of node-specific auxin flux, cytokinin supply, and strigolactone repression. ABA and ethylene act as stress-amplified switches that recalibrate underground expansion and branching patterns in response to environmental constraints (Rameau et al., 2019; Waadt et al., 2022). This conclusion is a cross-species generalization, and its practical implication for bamboo is a research prescription rather than a design specification: node-resolved quantification of indole-3-acetic acid, trans-zeatin, strigolactones and abscisic acid along an intact running rhizome, at dormant, activating and elongating nodes, is a prerequisite measurement that has not yet been reported for any bamboo, and without it the term ‘node-specific gradient’ remains qualitative (**Figure 4**).

**Figure 4:**
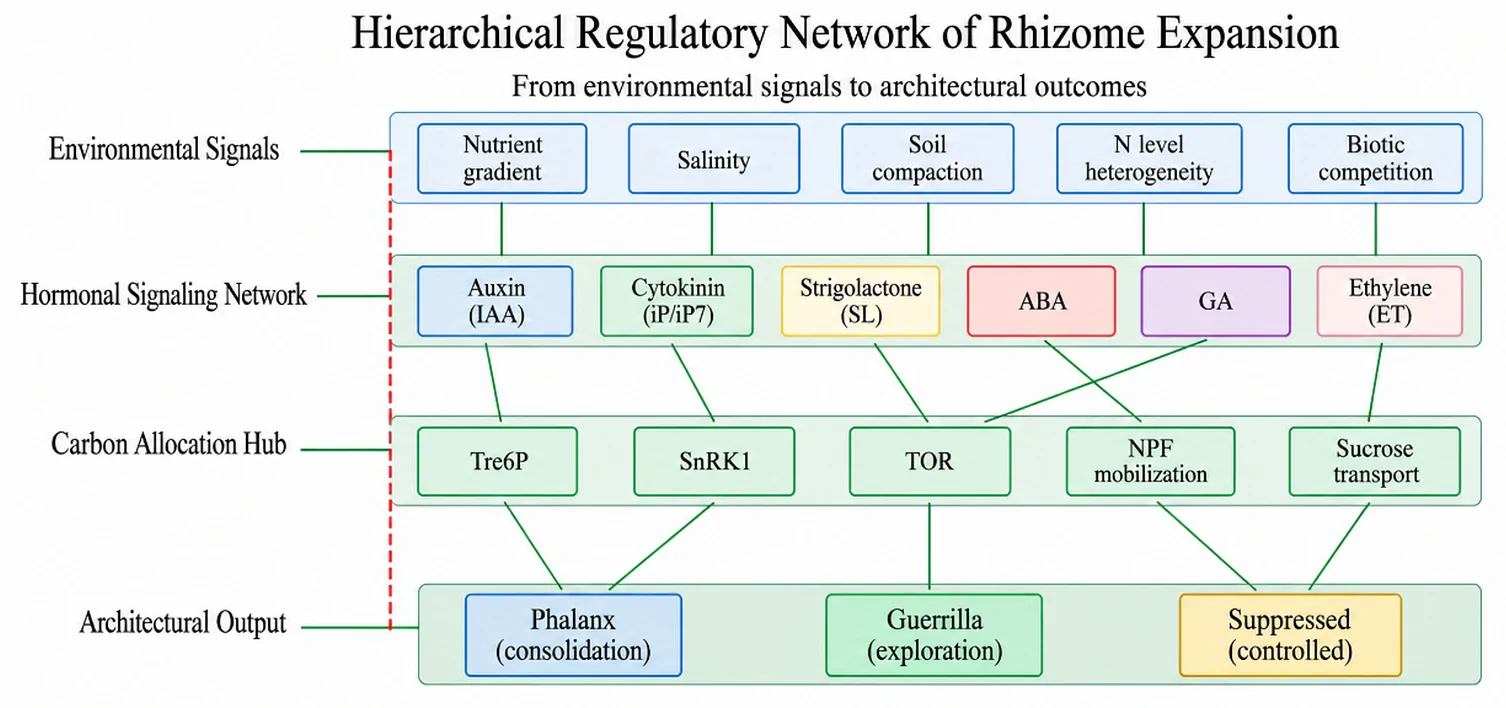
Hierarchical regulatory network of rhizome expansion in clonal plants. Environmental signals (Level 1) are transduced through hormonal signaling cascades (Level 2), integrating auxin, cytokinin, strigolactone, ABA, GA, and ethylene modules. These converge on carbon allocation hubs (Level 3), including the Tre6P -*SnRK1* -*TOR* axis and NSC mobilization, which determine the architectural outcome (Level 4): phalanx consolidation, guerrilla exploration, or controlled suppression. The red dashed feedback loop represents the environmental responsiveness of clonal integration. The cascade shown is assembled from causal evidence in Tier C systems and correlative evidence in bamboo; connections between Level 2 and Level 3 in particular have not been demonstrated in Bambusoideae and should be read as proposed rather than established (**Figure 1**, rows 9–18).

### 2.3 Carbon allocation networks and sugar signaling

It has been widely established that carbon allocation is not merely a passive outcome of photosynthetic supply but is actively regulated through sugar signaling networks. These networks integrate developmental stage and environmental status, determining the priority between growth, storage, and stress maintenance. Trehalose-6-phosphate (Tre6P) has been identified as a central signal metabolite linking sucrose availability to growth regulation. It forms a feedback nexus that adjusts sucrose synthesis, consumption, and starch remobilization across source and sink tissues (Figueroa and Lunn, 2016). Sucrose metabolism has been shown to act as both a metabolic gateway and signaling platform. It coordinates gene expression, hormonal crosstalk, and stress responses, influencing organ development and resource partitioning (Ruan, 2014). The antagonistic balance between *SnRK1* and *TOR* kinases has been described as a conserved growth-storage switch integrating carbon and nitrogen status. *SnRK1* generally promotes catabolic and survival pathways while repressing biosynthetic growth under carbon limitation (Persyn et al., 2024). Sucrose allocation can be engineered to enhance yield and drought resilience. This indicates that modification of carbon partitioning pathways directly alters productivity and stress tolerance (Griffiths and Paul, 2017). Starch has been characterized as a bifunctional carbon reservoir, acting dynamically as both source and sink. Its synthesis and degradation govern sugar availability and developmental transitions (MacNeill et al., 2017). The concept of plants functioning as an active carbon battery has been proposed. Starch-sugar interconversion is strategically regulated to buffer environmental fluctuations and sustain long-term growth (Zepeda Cabrera, 2023). The entire Tre6P -*SnRK1* -*TOR* framework summarized in this paragraph is Tier C in provenance: it was established in Arabidopsis, wheat, potato and yeast. Not one component of it has been genetically manipulated in any bamboo, and Tre6P itself has, to our knowledge, never been quantified in a bamboo rhizome. Its inclusion here is justified by the strong conservation of the pathway across angiosperms, but its application to bamboo is at present a Level 3 conceptual inference.

In relation to branching, rhizomatous extension, and sink activation, sugar has been demonstrated to function as a systemic regulator of bud outgrowth. It establishes sink strength through transporter induction, invertase activation, and starch remobilization (Doidy et al., 2024). In bamboo, rapid culm elongation has been shown to depend on non-structural carbohydrate (NSC) translocation from mature tissues to developing shoots. Coordinated regulation of sugar transport, cell division, and cell wall biosynthesis genes is involved (Chen et al., 2025). It is worth being precise about what this bamboo result does and does not show: NSC dynamics were characterized in the elongating culm and in the source tissues supplying it, not at rhizome apices or lateral buds, so the extension of the finding from culm elongation to rhizome extension is an inference across organs within the same species rather than a direct observation. Under drought in *Populus*, activation of the Tre6P/*SnRK1* pathway and shifts toward active sugar transport have been observed. Carbon repartitioning supports osmotic adjustment and survival rather than structural expansion (Fox et al., 2023). Similar metabolic reallocation toward survival has been reported in resurrection grasses. Energy metabolism tightly regulates programmed cell death and desiccation tolerance (Okemo, 2020). In wheat grain development under heat and drought, ABA-SnRK signaling interactions have been shown to modulate starch biosynthesis. Carbon flow toward storage tissues is actively reprioritized under stress (Ko et al., 2025). In lotus flower buds, reduced trehalose levels and altered *TPS1* signaling have been associated with bud abortion. This further supports the role of Tre6P-mediated sugar signaling in developmental fate decisions (Ren et al., 2022). Cross-species evidence has also indicated metabolic tradeoffs between carotenoids and carbohydrate pools. Carbon partitioning toward secondary metabolites can alter sugar and starch accumulation (Villwock et al., 2024). Collectively, across woody trees, cereals, bamboo, and horticultural species, carbon allocation has been repeatedly shown to be dynamically reset through sugar signaling hubs integrating Tre6P, *SnRK1*, *TOR*, hormone networks, and transporter systems (Sakr et al., 2018; Liu et al., 2025a; Burgess et al., 2022). Within this integrative framework, the regulation of horizontal expansion structures such as rhizomes or vigorous branching can be interpreted as a high-carbon-demand sink state maintained by elevated sucrose flux and suppressed stress signaling. Controlling diffusion may fundamentally involve reprogramming the Tre6P-*SnRK1* axis to reduce carbon allocation toward lateral expansion and redirect assimilates toward aboveground ornamental quality, structural reinforcement, or stress-defense maintenance. We retain this proposition but restate its status: it is a testable hypothesis (H3, Section 6) rather than a demonstrated mechanism, and the minimum experiment capable of discriminating it from the alternative that rhizome extension is limited by meristem competence rather than by carbon supply is a girdling or shading manipulation of individual connected ramets combined with isotopic carbon tracing to rhizome apices. By contrast, clonal integration and resource sharing among bamboo ramets is one of the few nodes in this review supported by Level 1 evidence in Tier A material, as demonstrated by isotopic tracing in running bamboo which has resolved nitrogen translocation as a function of rhizome branching architecture (Shi et al., 2021); experimental severing of rhizome connections has additionally shown that physiological integration is a manipulable control target in a related clonal grass (Otfinowski and Kenkel, 2008, Tier B).

## 3 Environmental plasticity and engineering management

### 3.1 Environmental plasticity and architectural modulation

Environmental modulation of root system architecture has been consistently interpreted as a plastic and potentially adaptive trait shaped by water gradients, nutrient heterogeneity, and mechanical impedance across diverse plant taxa. Soil compaction has been shown to restructure root system architecture by restricting elongation, altering branching angles, and promoting genotypic differences in penetration capacity. Architectural plasticity can enhance exploration under high mechanical resistance (Correa et al., 2019; Kolb et al., 2017; Xu et al., 2025). Under drought or pulsed water regimes, deeper primary rooting, increased specific root length, reduced xylem diameter, and rapid post-rewetting growth have been observed. Both morphological simplification and reversible growth dynamics contribute to dehydration avoidance and episodic recovery (Hanslin et al., 2019; Padilla et al., 2013; Comas et al., 2013; Jiang and Whalen, 2025). Long-term irrigation experiments in forest systems have demonstrated that biomass and production respond plastically to altered moisture regimes. Certain morphological traits remain conserved, implying selective sensitivity among root traits (Brunner et al., 2019). Nutrient heterogeneity has been repeatedly shown to induce localized root proliferation and enhanced foraging precision. Increased lateral root length and biomass allocation to nutrient-rich patches are evidenced. This includes legacy-enhanced foraging plasticity in clonal grasses (Li et al., 2021b; Lynch et al., 2021b). Root hairs and anatomical phenes have been confirmed to scale nutrient uptake efficiency under contrasting soil textures. This reinforces the functional coupling between morphology and exploitation capacity (Vetterlein et al., 2022; Lynch et al., 2021a). Broader syntheses have emphasized that root plasticity under drought, salinity, compaction, and biotic interaction is regulated by coordinated architectural, anatomical, and hormonal pathways. Its adaptive value depends on environmental predictability and stress cycling (Karlova et al., 2021; Shelden and Munns, 2023; El Amrani, 2023; Schneider and Lynch, 2020; Colombi et al., 2024; Lynch et al., 2021; Pierret et al., 2007; Fromm, 2019). Within this framework, plastic responses may be distinguished from elastic, reversible adjustments depending on energetic trade-offs and stress recurrence. This supports the conceptualization of diffusion control or spatial expansion as an environmentally responsive, condition-triggered trait rather than a constitutive growth program (Colombi et al., 2024). An important qualification applies to this entire literature when it is transferred to rhizome management: it concerns roots, whose function is absorption and anchorage, rather than rhizomes, which are stems with nodes, buds and intercalary meristems. Mechanical impedance, in particular, has been shown to restrict root elongation in Tier C crop systems, but the response of a bamboo rhizome apex to compacted soil — whether it deflects, arrests, or activates lateral buds — has not been quantified, and this is directly relevant to barrier design, since deflection geometry is the mechanism on which angled barrier installation depends (**Table 5**).

**Table 5:** Plant strategies and trade-offs in response to environmental stress.

Environmental stress	Observed responses/Trade-offs	Impact on expansion	Plant system/Model	References
Aeolian Disturbance	Under burial: increased ramet size; under erosion: increased ramet number; trade-off between growth and reproduction.	Expansion strategies adjust flexibly according to stress type.	Reed (Phragmites communis)	(Ba et al., 2023)
Salt Stress	Belowground bud bank expansion with constrained aboveground growth; trade-off between persistence and expansion.	Lateral expansion is limited to preserve clonal persistence.	Leymus chinensis	(Gao et al., 2025a)
Saline-Alkali Stress	Reduced rhizome and tiller production with strong physiological constraints.	Effective suppression of expansion.	Leymus chinensis	(Gao et al., 2025b)
Drought	Carbohydrate reserves in rhizomes are protected rather than abandoned.	Expansion pauses; rhizomes serve as survival insurance.	General Rhizomatous Plants	(Lubbe et al., 2023)
Resource Heterogeneity	Activation of guerrilla exploratory growth in high moisture/nutrient patches.	Conditional activation of expansion rather than continuous spread.	Wetland Plants; Grasses	(Liu et al., 2025b; Fry et al., 2018)
Long-term Grazing	Growth-defense trade-offs mediated by hormonal shifts; reduced growth-promoting hormones and increased defense compounds; enhanced root foraging plasticity.	Overall growth suppressed but exploratory plasticity enhanced.	Leymus chinensis	(Qu et al., 2022; Li et al., 2021b)
Clonal Integration	Integration buffers alkalinity stress by redistributing resources from donor to receiver ramets, with measurable cost to donors.	Overall survival maintained, but expansion may be limited by donor costs.	General	(Zhang et al., 2015b; Liu et al., 2016)

The tension between stress tolerance and clonal spread has repeatedly been shown to be governed by context-dependent trade-offs in rhizomatous and stoloniferous species. Growth, storage, and exploratory expansion are differentially prioritized under environmental stress. In rhizomatous species such as Phragmites communis, trade-offs between vegetative growth and reproduction have been documented under aeolian disturbance. Ramet size increases under burial but ramet number increases under erosion. This indicates flexible allocation between expansion and consolidation depending on stress type (Ba et al., 2023). Similar stress-induced reallocation toward belowground structures has been observed under saline conditions in Leymus chinensis. Underground bud banks expanded while upward growth was constrained. Clonal persistence may be buffered even when lateral extension is physiologically limited (Gao et al., 2025a). Under saline-alkali stress, reductions in rhizome and tiller production have been coupled with strong physiological constraints. Uncontrolled expansion can be suppressed by ionic imbalance and water-use disruption (Gao et al., 2025b). These two results are worth flagging as an apparent inconsistency rather than smoothing over, because they bear directly on whether salinity can be used as a management lever: in the same genus and under nominally comparable saline stress, bud bank expansion was reported in one study and rhizome and tiller suppression in the other. The most parsimonious reconciliation is that stress intensity and spatial homogeneity, not salinity per se, determine the direction of the response, which is consistent with the heterogeneity results discussed below; but the reconciliation is *post hoc*, and a dose- and heterogeneity-resolved experiment is required before saline amendment could be recommended as a containment tool. Importantly, drought does not necessarily trigger abandonment of rhizomes. Carbohydrate reserves are conserved and translocated to maintain rhizome integrity. Storage organs function as stress insurance rather than expendable expansion modules (Lubbe et al., 2023). Resource heterogeneity further modifies this balance. Plastic root and rhizome allocation toward moist patches has been stronger in fibrous and rhizomatous architectures than in tap-rooted systems. Exploratory growth can be selectively activated under spatially structured drought (Fry et al., 2018). Evidence from heterogeneous saline-alkali soils has shown that rhizome expansion can be promoted in patchy environments despite clipping. Homogeneous stress suppresses clonal spread. Spatial heterogeneity permits conditional activation of expansion strategies (Qu et al., 2023). Clonal integration has been demonstrated to buffer alkalinity stress by redistributing resources from original to subsequent ramets, with measurable cost to donor modules. A cost-benefit balance prevents expansion from undermining stress tolerance (Zhang et al., 2015b). Reviews have consistently emphasized that clonal integration enables labor division, resource sharing, and stress buffering. Local suppression of growth in stressed modules is allowed while maintaining whole-clone persistence (Liu et al., 2016; Yang et al., 2023; Bittebiere et al., 2020). Long-term grazing has induced growth-defense trade-offs mediated by hormonal shifts. Reductions in growth-promoting hormones and increases in stress-related compounds have constrained stature but enhanced resilience. Stress memory can reprogram allocation without eliminating clonal capacity (Qu et al., 2022). Enhanced root-foraging plasticity rather than increased nutrient absorbability has been detected in grazing-exposed clonal offspring. Exploratory plasticity can be strengthened while overall resource capture remains conservative (Li X et al., 2021). Spatial expansion strategies in *Phragmites australis* have shifted between phalanx and guerrilla architectures along degradation gradients. Reversible architectural modulation rather than irreversible expansion loss is revealed (Jiao et al., 2020). Marsh clonal plants have adopted acquisitive, guerrilla-like belowground strategies specifically under high moisture. This reinforces the environmental contingency of expansion modes (Liu et al., 2025b). Transgenerational clonal plasticity has demonstrated that prior warming exposure can alter aboveground-belowground allocation ratios across generations without exhausting rhizome reserves. Conditional allocation patterns may be epigenetically mediated (DuBois et al., 2020; Quan et al., 2024). Collectively, clonal plants rarely pursue expansion at the expense of stress tolerance per se. Architectural plasticity, integration, storage conservation, and stress memory jointly enable conditional or tissue-specific modulation of expansion. Thus, in the context of bamboo or other rhizomatous taxa, controllable diffusiveness should not be conceptualized as a fixed reduction in rhizome growth, but as an environmentally triggered or organ-specific regulatory strategy. This strategy preserves drought and salinity resilience under adverse conditions while allowing reversible exploratory expansion under favorable regimes. The phalanx–guerrilla plasticity on which this conclusion principally rests is a Tier B result, demonstrated repeatedly in Phragmites and other herbaceous clonal graminoids. Whether a woody bamboo can execute the same reversible switch is unresolved: the running and clumping habits are treated in the bamboo literature as fixed taxonomic characters rather than as endpoints of a plastic continuum, and no study has reported a running bamboo shifting toward phalanx-like consolidation under environmental treatment. This distinction matters because the entire premise of environmentally gated, reversible containment presupposes that the plastic range exists in bamboo.

### 3.2 Engineering and management strategies

It has been consistently shown that for running bamboos and other rhizomatous clonal plants, the most engineering-realistic containment baseline has been set by physical isolation plus routine surveillance rather than by any single material choice. Rhizome/root barriers made from HDPE-class geomembranes were generally treated as mechanically adequate. Field failure was disproportionately attributed to seam/joint leakage, corner detailing, shallow embedment, and most critically inspection/maintenance lapses. These lapses allowed undetected rhizome bridging and subsequent escape (Kotangale et al., 2025; Su et al., 2025). In extension-facing syntheses of practice, it has been repeatedly emphasized that barrier continuity (overlap/fastening at seams), intentional rhizome deflection geometry (angled installation that forces upward expression), and scheduled perimeter checks (with immediate cutting of surfaced rhizomes) were the determinants of long-term performance rather than nominal polymer type alone. This is, however, a qualitative attribution of failure mode rather than a quantitative reliability model. The reviewed sources report which failure mechanisms were observed but not the denominators required to convert them into design guidance: breach probability per meter of seam per year, the marginal reduction in breach probability per additional 0.1 m of embedment depth, or the inspection interval beyond which detection probability falls below a specified threshold are not reported in any source located by this review. Barrier specification therefore remains a matter of practitioner convention rather than of engineering reliability analysis, and Section 5.3 identifies this as the most tractable of the outstanding quantitative gaps, since it requires only systematic record-keeping rather than new biology. Repeated mechanical suppression (clipping/mowing/pruning) can reduce aboveground vigor across clonal invaders. Belowground reserves and spacer integration can sustain persistence unless disturbance is frequent and sustained. Trenching or rhizome severing has been treated as an effective but labor- and cost-intensive strategy. Its feasibility has been bounded by the need for repeated operations and by collateral impacts on root systems in managed landscapes (Ye et al., 2023; Zhai et al., 2022; Bam et al., 2026; Martin et al., 2020; Miesbauer et al., 2025). For bamboo specifically, the management objective function has been shown to be intrinsically multi-criteria. Shoot yield, culm production, and rapid clonal expansion are coupled through rhizome-mediated resource sharing and strong responsiveness to water and nutrient inputs. Irrigation and fertilization regimes have been repeatedly associated with increased shoot/culm productivity and altered biomass allocation. Deliberate water-fertility limitation can be used to reduce expansion pressure but would be expected to trade off with ornamental quality and production goals (Fang et al., 2019; Kim et al., 2018; Li et al., 2024). The magnitude of that trade-off is itself unquantified. No study located by this review reports paired measurements of expansion rate and shoot or culm yield across a water-and-nutrient gradient in the same stand, so the exchange rate between containment and production — how much yield must be forgone per unit reduction in lateral extension — cannot be stated. Any multi-objective optimization of the kind proposed in Section 4.4 is therefore currently unparameterized (**Table 6**).

**Table 6:** Summary of control strategies for rhizomatous plants.

Strategy category	Specific methods	Key success factors/mechanisms	Limitations/trade-offs	References	Provenance/causal level	Quantification status
Physical Isolation	HDPE Root Barrier Films	Seam sealing, adequate embedment depth, corner detailing, and regular inspection.	Installation quality and maintenance determine success, not material properties alone.	(Kotangale et al., 2025; Su et al., 2025)	A/2	Failure modes described qualitatively; no breach probability per metre of seam per year, and no effect size for embedment depth or inspection interval
Mechanical Suppression	Clipping/Trenching (Rhizome Severing)	Frequent and sustained operations to deplete belowground reserves.	Labor-intensive, high cost, collateral damage to landscape.	(Ye et al., 2023; Zhai et al., 2022; Miesbauer et al., 2025)	A-B/1	Efficacy demonstrated; required cutting frequency, duration to reserve depletion, and cost per unit boundary length not reported
Horticultural Management	Water and Nutrient Limitation	Reduced water and nutrient inputs decrease expansion pressure.	Clear trade-offs with ornamental quality and yield objectives.	(Fang et al., 2019; Kim et al., 2018; Li et al., 2024)	A/1	Direction established; no paired expansion-versus-yield measurements across a resource gradient, so the exchange rate is unknown
Ecological Engineering	Microbiome Management	Modulating rhizosphere/endosphere communities may influence rhizome vigor and nutrient acquisition.	Causal relationships require confirmation; considered promising medium-term strategy.	(Wang et al., 2025b; Zhang et al., 2022; Cui et al., 2021)	A/2	Observational only; no inoculation or suppression experiment has altered rhizome extension rate, and the direction of causality is unresolved
Genome Editing	Targeted Regulation	Editing key genes regulating rhizome development, hormone signaling, or resource allocation.	Provides incremental gain space as the ultimate solution after other methods.	(Wang et al., 2025a; Freschet et al., 2021)	C/3	No architectural editing has been performed in any bamboo; transformation efficiency for *Phyllostachys edulis* is not reported on a comparable basis (Section 4.2.1)

Provenance and causal-level codes are defined in Section 1.1 and in the note to **Table 4**.

It has also been increasingly supported that soil biotic context can shift rhizome health and expansion capacity. An intermediate microbiome management route has been justified as potentially lower-risk and nearer-term than genome editing. Convergent evidence shows that bamboo rhizosphere and endosphere communities (including AMF and diverse endophytic bacteria) are associated with nutrient acquisition, stress resilience, and potentially shoot initiation phenotypes. Long-term organic amendments and bamboo expansion gradients have been linked with measurable restructuring of rhizosphere/endophytic assemblages and soil enzyme-stoichiometric constraints. Belowground ecological engineering (substrate, amendment, inoculation, or suppression strategies) could plausibly modulate rhizome vigor and spread if causal levers are confirmed (Wang et al, 2025b; Zhang et al., 2022; Cui et al., 2021; Tong et al., 2025). The conditional in that last sentence carries the weight of the argument. All bamboo microbiome evidence located by this review is observational: communities differ between expanding and non-expanding stands, or between amended and unamended soils, but no inoculation or suppression experiment has demonstrated that a defined microbial manipulation changes rhizome extension rate. The direction of causality is unresolved, and the alternative interpretation — that altered rhizome physiology restructures the community rather than the reverse — is equally consistent with the published data. Microbiome intervention is accordingly presented here as a hypothesis-generating avenue, not as a control layer that can currently be specified. When these strands are taken together, a practical feasibility baseline has been delineated. Containment performance has been dominated by workmanship and inspection cadence at barrier seams/corners and by systematic perimeter root/rhizome pruning. Horticultural throttling has been mediated through predictable water-nutrient-allocation coupling but has required explicit trade-off framing against yield/ornamental targets. Microbiome-centric interventions have been positioned as a credible middle layer control concept. Rhizosphere/endosphere functions have been repeatedly correlated with bamboo productivity traits and with spread-associated soil process shifts. This clarifies the incremental gain space that would remain for genomics-based solutions after best-in-class non-transgenic controls have been implemented (Freschet et al., 2021; Kotangale, 2025; Wang, 2025).

## 4 Genomic regulation, editing, and comprehensive assessment

### 4.1 Comparative genomics and regulatory networks

The transition from uncontrolled rhizomatous spread to programmable underground architecture in bamboo must be grounded in comparative genomics and meristem-centered regulatory theory. Chromosome-level assemblies in hexaploid woody bamboos have recently revealed lineage-specific gene family expansions associated with root development and carbohydrate metabolism. Subgenome-biased expression and recent whole-genome duplication provide a genomic substrate for dissecting rhizome elongation and branching traits (Naktang et al., 2025). Polyploidy is not only an opportunity here but also the principal obstacle to editing: in a hexaploid genome a loss-of-function phenotype generally requires simultaneous inactivation of three homoeologous copies, which multiplies the required editing efficiency, increases the probability of off-target events, and makes the recovery of a complete null in a recalcitrant species substantially less likely. This is a further reason, independent of biosafety, to prefer dosage-modulating cis-regulatory edits over knockouts in Bambusoideae. Comparative and transcriptomic analyses in bamboo have further shown that conserved flowering and stress regulatory pathways can be resolved through homology-based and expression-based approaches. These include photoperiodic, hormonal, and drought-responsive networks. Key developmental switches are retrievable in Bambusoideae using cross-species frameworks (Biswas et al., 2016). Ecological-genetic evidence has demonstrated that running bamboos possess architectural and physiological attributes that confer significantly higher invasion risk than clumping species. Dense thicket formation and rhizome fragmentation capacity have been identified as decisive traits. Rhizome growth form is a selectable and evolutionarily differentiated phenotype (Lieurance et al., 2018). Field-based isotopic tracing in running bamboo has further revealed that rhizome branching architecture constrains nitrogen translocation. Source-sink dynamics are preferentially maintained within zero-barrier zones. Clonal integration and expansion intensity are structurally regulated rather than purely distance-dependent (Shi et al., 2021). Spatial genet analyses in dwarf bamboo have shown that productive genets expand directionally along light gradients and recolonize shaded microsites following die-off events. Clonal spread is environmentally responsive and genotype-dependent. Linking expansion vigor with stress adaptation is feasible (Tomimatsu et al., 2020). Complementary root distribution studies in *Bambusa arundinacea* have demonstrated that lateral rooting intensity declines predictably with distance and scales with clump size. Underground architecture is quantitatively modulated and amenable to management or genetic control (Mohan and Divakara, 2001).

At the meristematic and molecular levels, root apical meristem maintenance has been shown to depend on coordinated auxin-cytokinin crosstalk and transcription factor networks such as SHORTROOT-SCARECROW and PLETHORA. These collectively balance proliferation and differentiation, providing a conserved regulatory blueprint for rhizome tip activity (Sebastian and Lee, 2013). The conceptual framework of the quiescent center as a stress-resistant organizer maintaining stem cell niches has been expanded across model and crop species. Meristem resilience under abiotic stress is an intrinsic and regulatable property (Dubrovsky and Ivanov, 2021). Classical and developmental analyses have demonstrated that cell proliferation domains shift basally as roots elongate. Meristem expansion accompanies growth transitions. Spatial plasticity in proliferative zones could underlie differential rhizome internode elongation (Macleod, 1976; Clowes, 1959). In bamboo specifically, intercalary meristems have been identified as drivers of rapid internodal elongation. Culm and rhizome extension rely on specialized meristematic modules distinct from typical root systems (Xiong et al., 1980). This anatomical distinction deserves more emphasis than it usually receives, because it determines where an edit must act. Rhizome extension in bamboo is not driven by a root apical meristem but by an apical meristem at the rhizome tip together with intercalary meristems at the base of each internode; spacer length is therefore a product of intercalary cell division and expansion, which is precisely the process controlled by gibberellin metabolism. A promoter edit that reduces bioactive gibberellin locally in intercalary meristems would shorten spacers without necessarily affecting the number of buds produced, whereas an edit targeting bud outgrowth repressors would alter ramet density without altering spacer length. These are separable phenotypic axes and should be targeted separately rather than through a single ‘reduce expansion’ intervention. Zone-specific proteomic profiling under drought in switchgrass has demonstrated that root apex tissues preferentially activate cell proliferation and chromatin-modifying proteins. Elongation zones regulate cell wall remodeling. Stress tolerance and growth processes are differentially partitioned along the root axis yet remain coordinated during recovery (Ye et al., 2020). Molecular dissection of vegetative propagation in bulbous plants has further shown that axillary bud outgrowth and *de novo* meristem initiation are governed by conserved regulators such as *TB1/BRC1*, AP2/ERF factors, and hormone-mediated signaling. Branching intensity and meristem competence can be transcriptionally reprogrammed through integrated bottom-up and top-down approaches (Moreno-Pachón, 2017). Together, these findings suggest that bamboo rhizome expansion should be conceptualized as a modular meristem-driven system. Comparative genomics can identify candidate regulators. Spatiotemporal transcriptomics can resolve stress-expansion co-regulation. Population-level analyses can associate allelic variation with expansion distance and internode length. Multi-omics integration can pinpoint hub genes linking hormone flux, carbohydrate allocation, and meristem activity. The underground spread of bamboo may be repositioned from an uncontrolled wild trait to a genetically programmable horticultural phenotype. This phenotype retains clonal integration and stress resilience while constraining invasive expansion. The word ‘may’ in that final proposition is load-bearing, and Sections 4.2 and 5 are devoted to specifying what would have to be true for it to become ‘can’ (**Figure 5**).

**Figure 5:**
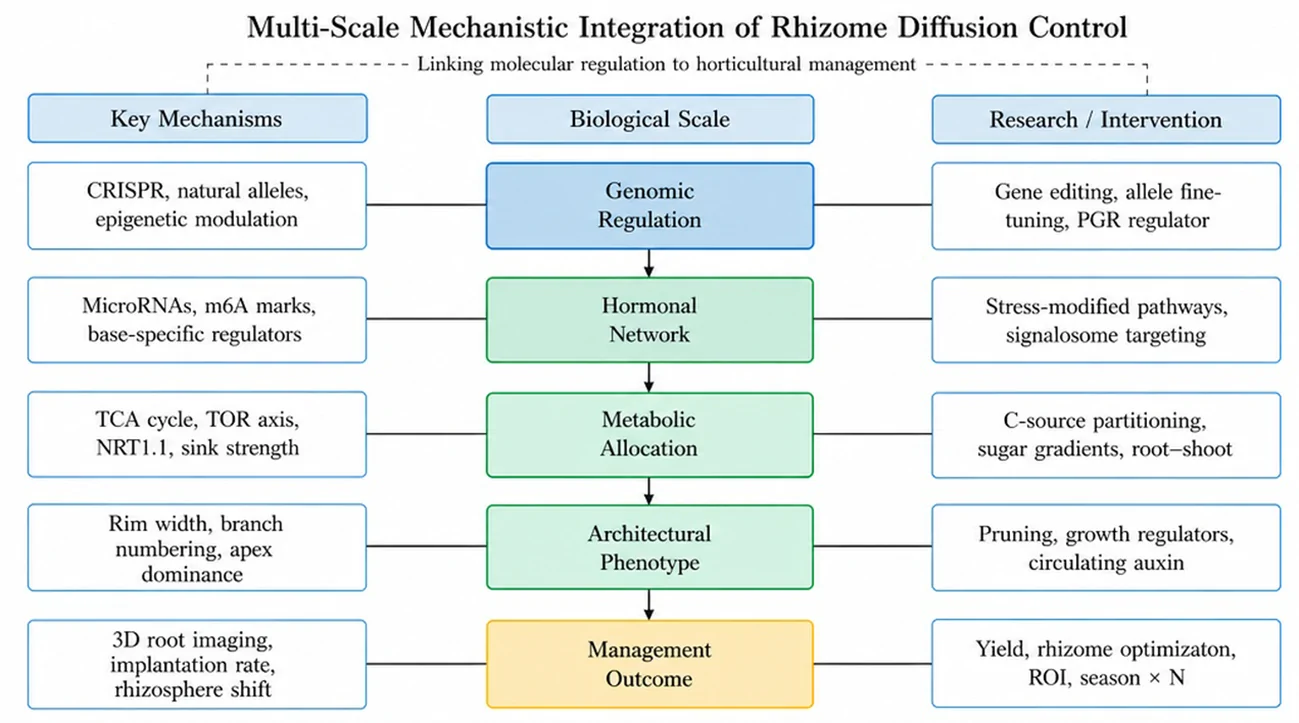
Multi-scale mechanistic integration framework for rhizome diffusion control. Five biological scales (center column) are linked through key mechanisms (left) and corresponding research or intervention approaches (right). Genomic regulation through CRISPR-based promoter editing cascades down to hormonal networks, metabolic allocation, and ultimately architectural phenotype, which informs management outcomes. This systems view supports the paradigm shift from reactive containment to programmable, multi-objective architectural management in bamboo and other clonal plants.

### 4.2 Genome editing strategies for architectural modulation

Recent advances in precise genome engineering have increasingly supported a tiered strategy for architectural modulation in plants. Low-risk, reversible, and tissue-specific interventions are prioritized over constitutive or knockout approaches. CRISPR-based systems can be deployed not only for gene disruption but also for promoter engineering, transcriptional activation/repression (CRISPRa/CRISPRi), base editing, and epigenetic modification. This enables graded and context-dependent modulation of gene expression rather than complete loss-of-function (Li et al., 2021a; Sathee et al., 2022; Min et al., 2022). In woody plants and perennial systems, developmental plasticity and long life cycles impose higher biosafety constraints. Targeted editing of regulatory regions and inducible or spatially restricted expression has been emphasized as a more controllable strategy to avoid growth collapse or pleiotropic penalties (Min et al., 2022). Evidence from rice and other crops has shown that promoter-level or tissue-directed manipulation of cytokinin catabolism genes can increase lateral branching without broadly compromising other agronomic traits. Regulatory fine-tuning is sufficient to reshape plant architecture (Wang et al., 2021). Amino acid transporter modulation in rice has been shown to enhance tillering and grain yield through coordinated effects on auxin, cytokinin, and strigolactone signaling. Moderate adjustments in nutrient allocation nodes can rebalance source-sink dynamics and branching intensity (Ji et al., 2020; Qin et al., 2023). The integration of miRNA-mediated nutrient signaling further supports the feasibility of subtle transcriptional reprogramming rather than structural gene ablation to maintain homeostasis under stress (Yang et al., 2024). These findings support the prioritization of regulatory-element editing and inducible systems as low-risk/high-controllability approaches that reduce the likelihood of systemic growth defects.

#### 4.2.1 Transformation and regeneration constraints specific to bamboo

Before candidate targets are discussed, the delivery problem must be stated plainly, because it determines which of the strategies above are actually available in Bambusoideae rather than in principle. Bamboo is among the more recalcitrant monocot groups for genetic transformation. Regeneration proceeds almost exclusively through somatic embryogenesis from embryogenic callus initiated on immature zygotic embryos, mature seeds or young inflorescence tissue, and the supply of such explants is itself constrained by the flowering behavior discussed below: in a species that flowers gregariously at multi-decadal intervals, seed-derived explants are available only in the years following a flowering event. Callus induction, embryogenic competence and plantlet regeneration are all strongly genotype-dependent, and reported transformation systems cluster in the tropical sympodial genera *Dendrocalamus* and *Bambusa* rather than in the temperate monopodial *Phyllostachys* taxa whose running rhizomes constitute the actual management problem. Reported efficiencies in the primary literature are seldom expressed on a comparable basis — some sources report the proportion of calli producing resistant lines, others the proportion of explants yielding a regenerated plantlet — and no meta-analyzable estimate of transformation efficiency for *Phyllostachys edulis* exists in the corpus reviewed here. This is itself a reportable finding: the parameter that most strongly conditions the feasibility of every proposal in this section is not currently quantified in a usable form.

Three practical consequences follow. First, the realistic near-term platform for functional work is a transformable sympodial background, with a validated phenotype subsequently transferred to a monopodial target only once the underlying biology is established; proposals that begin with editing in *Phyllostachys edulis* are, on present evidence, proposing the hardest possible entry point. Second, because regeneration passes through a callus phase with prolonged *in vitro* culture, somaclonal variation is a substantive confounder, and any architectural phenotype reported in a regenerated bamboo must be controlled against non-edited regenerants carried through the identical culture process rather than against seed-grown wild type. Third, and most consequentially, the conventional route to a transgene-free edited plant — segregating the editing construct away in a sexual generation — is unavailable in bamboo on any practical timescale. This single fact reorders the entire strategy space, and is developed in Section 4.2.4.

#### 4.2.2 Named candidate targets and their evidential status

Candidate targets can be organized by the phenotypic axis they are expected to move. For spacer and internode length, the gibberellin metabolic and signaling genes are the most defensible entry point: *GA2ox* genes, whose products deactivate bioactive gibberellins, *GA20ox* genes acting in the biosynthetic direction, and the *DELLA* gene *SLR1*. The rationale is that intercalary meristem elongation is the direct determinant of spacer length in bamboo (Section 4.1) and is gibberellin-dependent, and that *GA2ox* overexpression is the classical route to internode shortening in cereals. For ramet density and bud activation probability, the relevant targets are the bamboo orthologues of BRANCHED1/TB1 and the cytokinin oxidase/dehydrogenase (*CKX*) family, manipulated at the promoter rather than the coding level. For carbon partitioning to the expanding sink, *TPS1, SnRK1* subunit genes and hexokinase are candidates. It must be recorded that of these, only the gibberellin genes have any bamboo-specific functional support, and even that support is largely expression-based; the *BRC1/TB1* and *CKX* orthologues have been annotated in bamboo genomes but not functionally tested, and no *TPS1, SnRK1* or hexokinase gene has been manipulated in any bamboo.

The same caution applies to the microRNA layer, which is frequently proposed as a route to graded architectural control. The *miR156–SPL* module, the *miR319–TCP* module and *miR171–SCL* are all represented in bamboo small-RNA datasets and have been reported as differentially abundant between rhizome, shoot and culm tissues or across developmental stages. Their architectural functions, however, are established in rice, maize and Arabidopsis, not in Bambusoideae: to our knowledge no bamboo miRNA has been shown, by target mimicry, overexpression, knockdown or editing of its MIR locus, to alter rhizome architecture. The appropriate next step is therefore not to edit a MIR locus but to establish the regulon: degradome-supported target validation in rhizome tissue specifically, followed by comparison of small-RNA and degradome profiles between monopodial and sympodial species and between dormant and activating rhizome buds. Presenting bamboo miRNAs as ready editing targets overstates the evidence by at least one tier.

#### 4.2.3 Achieving spatially restricted regulation in an integrated clone

Spatial restriction is usually discussed as a promoter engineering problem, but in a clonal rhizomatous plant it has four distinguishable levels, and confusing them is a source of over-optimism. The first is transcriptional restriction, in which expression is confined to a tissue by a cell-type-preferential promoter; the required promoters — rhizome-apex, intercalary-meristem or rhizome-bud preferential — have not been characterized in bamboo, and identifying them from existing tissue-resolved transcriptomes is a prerequisite piece of work. The second is chemical inducibility, in which an applied ligand gates expression; this is attractive for reversibility but faces a delivery problem in a soil-buried organ, and any inducer must be shown to reach rhizome nodes at effective concentration. The third is environmental gating, in which an endogenous stress- or moisture-responsive promoter restricts expression to the conditions under which containment is actually needed. The fourth, and in our view the most robust, is molecular locality: choosing a target whose product does not move. A *GA2ox* enzyme deactivates gibberellin where it is expressed, so the phenotype is confined to the tissue by the biochemistry rather than by the promoter, whereas a transcription factor that alters systemic hormone or sugar status will produce effects wherever those signals travel.

This last consideration leads to a caveat that appears not to have been articulated in the rhizome engineering literature and that we regard as important. Clonal integration means that ramets connected by rhizomes exchange water, nitrogen and carbohydrate, and isotopic tracing in running bamboo has shown that this exchange is structured by rhizome branching architecture (Shi et al., 2021). Consequently, transcriptional restriction does not guarantee physiological restriction: an edit expressed only in rhizome tissue may still alter the source–sink status of the whole genet and thereby change the growth of unedited connected ramets. Demonstrating spatial specificity by promoter–reporter assay is therefore insufficient. Specificity must be verified at the level of the integrated clone, by carbon and nitrogen isotopic tracing between connected edited and unedited ramets, and by phenotyping ramets at increasing rhizome distance from the site of expression. We recommend that this be treated as a mandatory validation step rather than an optional refinement.

#### 4.2.4 Which strategy is realistically achievable in bamboo

Ranking the available strategies against the constraints set out above produces a clear and somewhat counter-intuitive ordering. Multiplexed constitutive knockout of core developmental genes, although it produces the largest architectural effects reported in any system, is the least appropriate option for bamboo: hexaploidy requires simultaneous inactivation of homologues, the effect is irreversible in a plant that persists as a clone for decades, strigolactone pathway loss carries documented penalties for drought tolerance and arbuscular mycorrhizal symbiosis, and the editing construct cannot be removed. CRISPRa and CRISPRi are valuable but must be classified honestly: because the dCas9 effector has to remain present for the effect to persist, a CRISPRa- or CRISPRi-modified bamboo is necessarily and permanently transgenic. They are therefore excellent tools for functional validation and poor candidates for deployment. Base and prime editing occupy an intermediate position, offering precise hypomorphic alleles without double-strand breaks, but they require higher delivery efficiency than is currently reported for bamboo.

Among the available strategies, delivery of preassembled Cas9 or Cas12a ribonucleoprotein complexes into protoplasts or embryogenic callus, targeting cis-regulatory elements, is most consistent with all four constraints. Ribonucleoprotein delivery leaves no integrated DNA, so the resulting plant is transgene-free at the moment of regeneration and does not require a sexual generation to become so; given current technology and the constraints of monocarpic flowering, this is the most practical route by which a transgene-free edited bamboo could be obtained within a realistic timeframe; future advances in alternative delivery systems or inducible reproductive systems might offer additional pathways, but none has been reported for bamboo at present. Targeting cis-regulatory elements rather than coding sequence yields dosage changes rather than nulls, which is both safer in a polyploid and more compatible with the graded, reversible control that the ecological argument requires. Our specific near-term recommendation is therefore ribonucleoprotein-delivered promoter editing of a gibberellin metabolism gene, or of a bud-outgrowth repressor, in a transformable *Dendrocalamus* or *Bambusa* background, with the phenotype validated first in protoplast and composite-plant systems and, where the orthologue permits, in rice or Brachypodium. The three tiers, their targets, their controllability and risk profiles, and their feasibility under bamboo-specific constraints are summarized in **Figure 2**; **Table 2**.

At the intermediate tier, stronger architectural effects have been achieved through modulation of hormone signaling hubs that integrate auxin, strigolactone, cytokinin, sugar, and light pathways. BRANCHED1 (*BRC1/TB1*) has been characterized as a central transcriptional hub integrating endogenous and environmental signals to suppress bud outgrowth. It is a sensitive node for architectural engineering (Wang et al., 2019; Leduc et al., 2014). In apple and Arabidopsis-related systems, strigolactone-mediated repression of adventitious rooting and branching has been mechanistically linked to SMXL-WRKY-BRC1 cascades. Direct promoter binding events have been identified as key regulatory points. Promoter editing or partial attenuation of these components could achieve graded suppression rather than binary inhibition (Fan et al., 2022; Fichtner et al., 2024). In poplar, attenuation of auxin-strigolactone signaling via PagKNAT2/6b has been shown to enhance branching following apical injury. Architectural plasticity can be modulated through hormone cross-talk nodes (Zhao et al., 2024). The poplar result is the only Level 1 causal demonstration of *KNOX*-mediated architectural control in a woody perennial, and poplar is a Tier C system for present purposes: it is a tree, not a rhizomatous clonal plant, and the phenotype concerns aerial branching after decapitation rather than belowground spacer or bud behavior. The bamboo *KNAT2/6* orthologues have not been functionally characterized. In cereals, CRISPR-mediated alteration of *NINJA*-domain proteins and *TFL1* homologs has resulted in marked changes in tiller number and shoot architecture. Stronger phenotypic shifts can be obtained by targeting central developmental regulators, albeit with detectable impacts on overall plant stature and reproductive traits (Hong et al., 2020; Sun et al., 2023). Parallel architectural compaction strategies in Nicotiana via *CCD7/CCD8* knockout have reduced plant spatial footprint by nearly half through strigolactone depletion. Careful evaluation of metabolic flux and hormonal balance was required. This underscores the higher systemic risk associated with core pathway disruption (Giroux et al., 2025; Naz et al., 2025). Metabolic reallocation through hexokinase editing in rice has demonstrated that carbon signaling nodes can substantially enhance photosynthetic efficiency and yield. Sugar signaling is tightly coupled to architectural outcomes and resource partitioning (Zheng et al., 2021). Taken together, cross-species evidence from annual crops, woody perennials, and controlled-environment systems indicates that architectural engineering can be stratified according to intervention depth. Regulatory-element editing and inducible/tissue-specific systems offer high controllability with reduced systemic penalties. Modulation of hormone and carbon signaling hubs yields stronger phenotypes but requires precise quantitative tuning. Direct disruption of core developmental or rhizome-forming factors may induce profound architectural suppression at the expense of vigor, resilience, or regenerative capacity. A conservative but bold strategy grounded in promoter editing, spatial restriction, and graded hormonal rebalancing appears to be the most biologically rational pathway for safely constraining expansion while preserving overall plant fitness. We would add only that ‘biologically rational’ and ‘currently executable’ are different claims, and that in bamboo the gap between them is presently defined by delivery efficiency and by the absence of characterized rhizome-preferential promoters rather than by any shortage of plausible targets.

### 4.3 Ecological and biosafety assessment

The ecological and biosafety implications of genetically modified perennial and ornamental species have been extensively examined in the broader context of GM crops, trees, and floricultural plants. These insights provide a rigorous comparative framework for evaluating edited bamboo systems. Horizontal gene transfer from GM plants to microorganisms occurs at extremely low frequencies. It is unlikely to pose measurable environmental risk under natural conditions (Keese, 2008; Pontiroli et al., 2007). Plant-to-microbe gene escape is generally negligible relative to background genetic exchange processes. Greater concern has been associated with vertical gene flow and persistence, particularly in long-lived or outcrossing species. Gene flow to compatible relatives and enhanced invasiveness must be assessed relative to conventionally bred comparators (Conner et al., 2003; Flachowsky et al., 2010). Assessment is inherently case-by-case and exposure-dependent. In woody perennials and trees, temporal scale, landscape connectivity, and reproductive biology have been highlighted as critical modifiers of risk. Long life cycles and vegetative propagation alter exposure dynamics compared with annual crops (Häggman et al., 2013). In floricultural plants, the proximity of wild relatives and ornamental escape history has been identified as a more significant determinant of gene flow risk than the transformation method itself (Kikuchi et al., 2008; Chandler and Sanchez, 2012).

#### 4.3.1 Why vegetative containment dominates reproductive containment in bamboo

The biosafety literature summarized above was developed largely for outcrossing annual crops and for trees that flower regularly, and in those systems reproductive containment through induced sterility is the canonical mitigation. Bamboo inverts the problem. Most woody bamboos are monocarpic and flower gregariously at intervals that in *Phyllostachys edulis* are conventionally given as several decades, after which the flowering cohort dies. Three consequences follow, and together they determine the risk architecture of any edited bamboo.

First, the realized pathway of spread and persistence is overwhelmingly vegetative. A running bamboo genet expands by rhizome extension and persists clonally between flowering events, so propagule pressure in the ecologically relevant sense is generated by rhizome fragments and by lateral extension across boundaries, not by seed. An edit that reduced fertility would leave the actual invasion mechanism untouched; conversely, an edit that reduced lateral extension or fragment regeneration would act directly on it. This is the reverse of the priority ordering assumed in most genetically modified tree risk assessments, and it should be stated explicitly in any dossier for an edited bamboo. Second, the same flowering biology makes sterility engineering practically unverifiable: a sterility phenotype could not be confirmed until the cohort flowered, which may be decades after deployment, so sterility cannot serve as a pre-release containment guarantee even if it were the relevant one. Third, and as noted in Section 4.2.4, the absence of an accessible sexual generation removes segregation as a route to transgene removal, which is why ribonucleoprotein delivery is not merely preferable but effectively obligatory for any material intended for open deployment.

The practical implication is that the risk assessment endpoints for edited bamboo should be reweighted rather than merely inherited. The dominant endpoints are the minimum rhizome fragment size capable of regeneration and its seasonal variation; the lateral extension rate of the edited genet relative to unedited controls under common conditions; the persistence of edited rhizome tissue in soil after removal; and the behavior of the edited genet under the disturbance regimes typical of the intended deployment setting, since fragmentation by cultivation or excavation is the principal amplification mechanism. Gene flow to compatible relatives, while not negligible, is a lower-priority endpoint on the timescales over which deployment decisions must be made. A staged deployment pathway from containerized and confined cultivation through semi-open landscape trials, with rhizome-directed monitoring at each stage, follows naturally from this reweighting and is incorporated into the roadmap in Section 4.5.

The mitigation of invasiveness through genetic sterility or reduced fertility has been proposed as a biologically meaningful containment strategy in woody ornamentals even when sterility is incomplete. Propagule pressure strongly determines establishment probability (Vining et al., 2012). Engineered modulation of reproductive or rhizomatous expansion in bamboo could similarly reduce ecological risk if effective containment thresholds are achieved. For traits conferring abiotic stress tolerance, environmental risk depends primarily on phenotype magnitude and receiving environment. Enhanced stress tolerance does not inherently translate into weediness without ecological opportunity (Khan et al., 2016). Niche shift assessments must quantify fitness effects under realistic community contexts. Plastid transformation has been discussed as offering improved transgene containment due to maternal inheritance in most angiosperms. Biosafety and public concerns remain subject to regulatory scrutiny (Wani et al., 2015). Plastid transformation, it should be noted, offers little practical advantage in this context: it addresses pollen-mediated transgene flow, which is not the operative pathway in a clonally spreading monocarpic species, and no plastid transformation system has been reported for any bamboo. Genome editing in conservation contexts has been characterized as a technology requiring cautious deployment under strict ecological justification (Yin et al., 2024). Reversibility, stability, and long-term monitoring are central to governance of edited perennials. Regulatory analyses have indicated that environmental risk assessment frameworks for GM and genome-edited plants converge on persistence, invasiveness, gene transfer, non-target interactions, and long-term effects as core endpoints (Schiemann et al., 2019; Alalwani et al., 2024). Public acceptance depends less on technical risk magnitude and more on transparency, regulatory trust, and perceived application domain. Ornamental GM plants face high regulatory costs despite documented market acceptance (Verma, 2013; Chandler and Sanchez, 2012). Collectively, ecological risk associated with modified perennial ornamentals is governed less by the editing technology per se than by life-history traits, reproductive containment, propagule pressure, and management context. A graded deployment pathway from confined cultivation to semi-open landscapes aligns with established ERA paradigms and may mitigate societal concern when supported by transparent, case-specific data (**Table 7**).

**Table 7:** Ecological and biosafety considerations for genome-edited perennial plants.

Risk category	Key findings/consensus	Implications for genome-edited bamboo	References	Endpoint priority for a clonally spreading, monocarpic species
Horizontal Gene Transfer	Transfer from transgenic plants to microorganisms occurs at extremely low frequencies; environmental risk is negligible.	Risk of edited DNA transfer to soil microorganisms is extremely low.	(Keese, 2008; Pontiroli et al., 2007)	Low priority. Not applicable at all to ribonucleoprotein-edited material, which contains no introduced DNA
Vertical Gene Flow	Primary concerns involve hybridization with wild relatives; risk assessment is case-dependent.	Bamboo has long flowering cycles, but hybridization potential with native species requires evaluation.	(Conner et al., 2003; Häggman et al., 2013)	Lower priority on deployment timescales. Gene flow requires a flowering event that may not occur for decades, whereas rhizome spread is continuous
Invasiveness/Persistence	Enhanced stress tolerance does not inherently translate to weediness; evaluation required in ecological contexts.	Competitiveness and persistence of modified bamboo in semi-natural environments must be assessed.	(Khan et al., 2016; Flachowsky et al., 2010)	Highest priority. Measured as lateral extension rate relative to unedited controls, minimum regenerative fragment size and its seasonal variation, and persistence of rhizome tissue in soil after removal
Genetic Containment	Engineering sterility or reduced fertility is an effective containment strategy even if incomplete.	Reducing rhizome expansion itself constitutes physical containment, lowering ecological risk.	(Vining et al., 2012)	Priority reversed relative to annual crops and regularly flowering trees. Sterility is neither the operative pathway nor verifiable before flowering; vegetative containment is the substantive endpoint (Section 4.3.1)
Technology and Regulation	Plastid transformation offers improved containment due to maternal inheritance; regulatory frameworks focus on persistence and gene flow endpoints.	Plastid editing or genetic containment technologies can be considered in design.	(Wani et al., 2015; Schiemann et al., 2019)	Low practical relevance. Plastid transformation addresses pollen-mediated flow, which is not the operative pathway, and no plastid transformation system exists for any bamboo
Public Acceptance	Depends on transparency, regulatory trust, and application domain rather than technical risk magnitude alone.	Special attention to public communication and transparency needed for ornamental applications.	(Verma, 2013; Chandler and Sanchez, 2012)	Moderate priority. The transgene-free status of ribonucleoprotein-edited material is the single most communicable feature and should be documented explicitly

### 4.4 Comprehensive evaluation framework

A rigorous evaluation framework for controllable rhizome diffusion must integrate underground phenotyping, mechanical validation, stress-response assessment, and multi-objective performance indices. This transforms conceptual design into quantifiable horticultural criteria. Three-dimensional tracking of rhizome architecture has been implicitly supported by architectural analyses demonstrating that branching angles, spacer length, and meristem deployment follow predictable spatial rules. These can be reconstructed through layered excavation or spatial modeling approaches (Bell and Tomlinson, 1980). Empirical analyses of clonal module variation in *Phragmites australis* along environmental gradients quantified internode length, branch angle, and ramet density. Temporal monitoring of rhizome expansion velocity is feasible and ecologically informative (Jiao et al., 2020). The ecology of belowground bud banks further emphasized that bud natality, dormancy, and depletion rates can be quantified to estimate regenerative potential. Measurable underground phenotypic parameters are provided for time-series analysis (Ott et al., 2019). In rhizomatous grasses such as Kentucky bluegrass, correlations between rhizome number, dry mass, and metabolic enzyme activities have demonstrated that physiological and structural traits can be integrated to evaluate expansion intensity (Ran et al., 2023). Systems-wide profiling of rhizome-specific genes and metabolites in *Oryza longistaminata* suggested that molecular phenotypes can be aligned with architectural measurements to build multi-layered expansion indices (He et al., 2014). Each of these precedents establishes that the corresponding measurement is feasible; none of them provides the bamboo reference values against which an edited genotype would be compared. Constructing that baseline — spacer length, branching angle, bud density and annual lateral extension, each with a dispersion estimate, for a defined species, age class and site type — is a prerequisite for the framework proposed here and is placed first in the roadmap of Section 4.5 for that reason.

Mechanical and engineering validation has been indirectly informed by evidence that clonal integration disruption reduces horizontal proliferation. This provides an experimental analog for trenching or root-barrier interventions. Estimation of overrun probability and maintenance frequency is enabled (Otfinowski and Kenkel, 2008; Liu et al., 2016). Studies on invasive *Polygonum cuspidatum* and *Butomus umbellatus* have demonstrated that vegetative fragments and incomplete rhizome removal lead to re-establishment. Engineering failure rates depend more on seam leakage, incomplete isolation, and maintenance gaps than on material properties alone (Barney et al., 2006; Cahoon, 2018). Stress-resilience verification has been supported by genomic and physiological evidence showing that rhizome development intersects with ABA-GA balance, carbohydrate remobilization, and stress signaling networks. Survival, post-stress recovery, photosynthetic efficiency, and root vitality must be co-evaluated alongside diffusion suppression (Huang et al., 2025; Chen et al., 2025; Wang et al., 2025a). Polyploidization-enhanced rhizome biomass allocation in *Solidago canadensis* demonstrated that increased expansion capacity can coincide with environmental robustness. Multi-trait evaluation is essential to avoid inadvertently selecting for either excessive invasiveness or reduced resilience (Feng et al., 2022). Microbial diversity in bamboo ecosystems has been linked to nutrient acquisition and stress tolerance. Rhizosphere context should be incorporated into comprehensive performance metrics (Wang et al., 2025b). These findings support the construction of an integrated evaluation system. Underground phenotypic tracking (three-dimensional rhizome architecture and expansion velocity), boundary overrun probability under engineered containment, abiotic stress survival and recovery parameters, and aboveground landscape or material performance are synthesized into a multi-objective index. This framework enables diffusion control to be quantitatively balanced against ornamental value, shoot yield, structural properties, resilience, cost, and maintenance frequency. Rhizome management is transformed from reactive containment into evidence-based horticultural design optimization. We emphasize that the index is at present a structure without coefficients. Weighting the objectives against one another requires the expansion-versus-yield exchange rate (Section 3.2) and the breach-probability function (Section 5.3), neither of which has been measured. Presenting the framework as immediately operational would misrepresent it; its present function is to specify which measurements must be made for a multi-objective optimization to become possible (**Table 8**).

**Table 8:** Components of the comprehensive evaluation framework for controllable bamboo rhizome diffusion.

Evaluation dimension	Key quantitative indicators	Theoretical basis/purpose	References	Current measurement status in bamboo
Belowground Phenotype Tracking	3D rhizome architecture, expansion velocity, internode length, branching angles, bud bank density.	Direct quantification of diffusion behavior and regenerative potential.	(Bell and Tomlinson, 1980; Jiao et al., 2020; Ott et al., 2019)	Method established in other clonal taxa. No bamboo baseline reporting these parameters with dispersion estimates by age class and site type has been located (Gap 1, Section 5.3)
Engineering Containment Validation	Boundary breakthrough probability, maintenance frequency, fragment regeneration rate.	Assessment of failure risk under real-world engineering constraints.	(Otfinowski and Kenkel, 2008; Barney et al., 2006; Cahoon, 2018)	Not quantified. Breach probability as a function of embedment depth, seam treatment and inspection interval is unreported, as is the minimum regenerative fragment size for any bamboo (Gaps 2 and 4, Section 5.3)
Stress Resilience Assessment	Survival rate, recovery rate, photosynthetic efficiency after drought/salt stress.	Ensuring diffusion control does not compromise core survival capacity.	(Huang et al., 2025; Wang et al., 2025a; Feng et al., 2022)	Measurable with standard methods; must be applied to edited versus tissue-culture regenerant controls rather than seed-grown wild type (Section 4.2.1)
Horticultural/Material Performance	Ornamental traits (leaf color, culm morphology), bamboo mechanical properties, shoot yield.	Multi-objective performance evaluation to ensure primary values are preserved or enhanced.	(Ran et al., 2023; Chen et al., 2025)	Individually measurable, but the expansion-versus-yield exchange rate needed to weight this dimension against containment has not been determined (Gap 5, Section 5.3)
Microbial Interactions	Rhizosphere microbial diversity, abundance of nutrient cycling-related functional genes.	Assessment of potential impacts on beneficial soil ecosystems.	(Wang et al., 2025b)	Measurable. Arbuscular mycorrhizal colonisation should be added as a specific non-target endpoint wherever a strigolactone-pathway target is contemplated

The framework is at present structurally specified but numerically unparameterized: weighting the objectives against one another requires the quantities listed in Section 5.3.

### 4.5 A staged experimental roadmap with explicit decision criteria

The preceding sections identify what is not known. This section converts those gaps into an ordered program in which each stage produces a decision rather than merely a dataset, and in which failure at an early stage prevents wasted investment at a later one. Four stages are proposed, with indicative durations that reflect bamboo’s growth rhythm rather than a funding cycle. The full specification, including outputs and go/no-go criteria, is given in **Table 3**.

Stage 1, baseline quantification and target nomination, requires no transformation capability and should proceed immediately. Its components are a rhizome architectural census in a defined running species, reporting spacer length, branching angle, bud density and annual lateral extension with dispersion estimates across age classes and site types; node-resolved hormone quantification along intact rhizomes at dormant, activating and elongating nodes; tissue-resolved transcriptome and small-RNA sequencing of rhizome apex, intercalary meristem, dormant bud and activating bud, from which rhizome-preferential promoter candidates are nominated; and a retrospective compilation of barrier installation records to estimate breach probability against embedment depth, seam treatment and inspection interval. The decision criterion for proceeding is that at least one candidate promoter shows a rhizome-preferential expression ratio sufficient for use and that hormone gradients along the rhizome are resolvable above measurement noise; if hormone concentrations do not differ detectably between dormant and activating nodes, the hormonal gating model as applied to bamboo is not supported and the program should redirect toward carbon and meristem-competence explanations.

Stage 2, causal validation in surrogate systems, tests the mechanism before committing to bamboo transformation. Candidate genes nominated in Stage 1 are functionally tested by transient expression and CRISPRi in bamboo protoplasts, by Agrobacterium rhizogenes composite plants where the species permits, and by orthologue editing in rice or Brachypodium where the orthology relationship is unambiguous; pharmacological perturbation of intact bamboo rhizomes with gibberellin, gibberellin biosynthesis inhibitors, cytokinin and synthetic strigolactone analogues provides the first causal test in Tier A material and is, notably, achievable without any transformation. The decision criterion is that pharmacological perturbation produces the predicted directional change in bud activation or internode elongation in bamboo itself; a target that fails this test should not be carried forward regardless of how well it performs in the surrogate systems, because failure indicates that the module is not rate-limiting in bamboo.

Stage 3, editing and containment-relevant phenotyping, is the first stage requiring transformation. It comprises ribonucleoprotein delivery of a validated cis-regulatory edit into embryogenic callus of a transformable *Dendrocalamus* or *Bambusa* background, regeneration with non-edited tissue-culture regenerants as the control class, molecular confirmation of the edit and of the absence of integrated DNA, and phenotyping in containerized and rhizotron systems over at least two full growth cycles. The phenotype set must include not only spacer length and bud activation but also the containment-relevant endpoints identified in Section 4.3.1: minimum regenerative fragment size, fragment regeneration probability by season, and lateral extension rate relative to controls. Spatial restriction must be verified by carbon and nitrogen isotopic tracing between connected edited and unedited ramets, not by reporter localization alone. The decision criterion is a statistically supported reduction in lateral extension accompanied by no detectable penalty in drought and cold survival, photosynthetic performance, or culm mechanical properties; a phenotype that reduces expansion only by reducing overall vigor has not solved the problem posed by this review.

Stage 4, confined field evaluation and transfer, proceeds only if Stage 3 criteria are met. It comprises multi-year barriered field trials with rhizome-directed monitoring, quantification of the expansion-versus-yield exchange rate across a water and nutrient gradient in edited and unedited material, assessment of rhizosphere community and mycorrhizal colonization as non-target endpoints, and, in parallel, the attempt to transfer the validated cis-regulatory edit into a monopodial *Phyllostachys* background as transformation capability in that genus improves. Regulatory engagement should begin at Stage 3 rather than Stage 4, since the dossier requirements for a clonally propagated, transgene-free edited perennial are not settled in most jurisdictions and will shape the phenotyping design.

## 5 Evidence provenance, causal strength, and outstanding knowledge gaps

### 5.1 Where the evidence actually comes from

The mechanistic account assembled in Sections 2 to 4 is coherent, but coherence is not the same as validation, and a reader is entitled to know which parts of it rest on experiments in bamboo, which on experiments in other clonal plants, and which on inference from annual model systems. We therefore scored every regulatory and management node invoked in this review against the two-axis scheme defined in Section 1.1 — provenance tier and causal level — using only the reference list of this review as the evidence base. Twenty-nine nodes were scored across seven domains: rhizome architecture, the belowground bud bank, hormonal regulation, carbon allocation, environmental modulation, engineering and management, and genomic regulation and editing. The full matrix is shown in **Figure 1** and reported node by node in **Table 1**.

Three features of the matrix deserve comment. First, causal evidence in woody bamboo is rare. Only two of the twenty-nine nodes carry Level 1 evidence obtained in Bambusoideae: the distinction between running and clumping rhizome habit as a determinant of expansion and invasion risk, and clonal integration together with architecture-structured resource sharing, the latter demonstrated both by isotopic tracing in running bamboo and, at the level of management rather than molecular biology, by the experimental severing of rhizome connections. Every other bamboo entry is Level 2 or Level 3. Second, the density of Level 1 evidence is highest in precisely the columns least transferable to the target problem: the strigolactone, *BRC1/TB1*, *KNOX* and Tre6P -*SnRK1* -TOR nodes are supported by strong causal work in Arabidopsis, rice, Nicotiana and poplar, and by nothing in bamboo. Third, the environmental and bud-bank domains are anchored in Tier B clonal graminoids — Phragmites, Leymus, Solidago, Poa — where the evidence is often both causal and quantitative, but where the growth form is herbaceous and the rhizome is not woody.

The practical reading of this pattern is not that the framework is unfounded, but that its load-bearing assumption is unexamined. The framework assumes that the auxin–cytokinin–strigolactone module and the sugar-signaling module operate in a woody bamboo rhizome as they do in the aerial axillary buds of annual grasses. That assumption is plausible on orthology grounds and is not contradicted by any bamboo dataset, but it has not been tested, and a program that proceeds directly to editing is betting the entire investment on it. The pharmacological perturbation experiment placed in Stage 2 of the roadmap (Section 4.5) exists specifically to test this assumption cheaply and early, since it requires no transformation.

### 5.2 Conflicting evidence and the boundary conditions of the framework

A review that reports only convergent findings misrepresents its literature. Four points of genuine tension emerged during the appraisal and are recorded here because each defines a boundary condition on the proposals made above.

The first concerns the direction of the salinity response. In *Leymus chinensis*, saline stress has been reported both to expand the belowground bud bank and to suppress rhizome and tiller production. Both cannot be general. The reconciliation we favor — that intensity and spatial heterogeneity, rather than salinity as such, set the direction — is consistent with the heterogeneous saline-alkali results in which patchiness promoted rhizome expansion while homogeneous stress suppressed it, but it is a *post hoc* reading of independent experiments. Until a dose-resolved and heterogeneity-resolved experiment is performed, edaphic amendment cannot be recommended as a containment lever, because a sub-threshold application could plausibly increase rather than decrease bud production.

The second concerns whether expansion and stress tolerance genuinely trade off. Section 3.1 develops the argument that clonal plants rarely sacrifice tolerance for spread, and the integration and storage-conservation literature supports this. The polyploidization work in *Solidago canadensis* points the other way: increased rhizome biomass allocation coincided with environmental robustness rather than trading against it. If expansion capacity and resilience can rise together, then an intervention that reduces expansion may not automatically preserve resilience as a by-product, and the co-evaluation of stress endpoints demanded in Section 4.4 becomes obligatory rather than precautionary.

The third concerns the transferability of the phalanx–guerrilla model. That model is a Tier B generalization from herbaceous clonal graminoids in which architecture is demonstrably plastic. In bamboo, running and clumping habits are treated as fixed taxonomic characters associated with monopodial and sympodial rhizome systems, and no experimental demonstration of a reversible shift within a running species has been located. The entire proposition of environmentally gated, reversible containment presupposes that a plastic range exists in bamboo. If the running habit proves to be canalized, then only constitutive modification would be effective, and the risk calculus of Section 4.2 would shift substantially toward caution.

The fourth concerns hormonal hierarchy in bud release. The literature variously assigns primacy to auxin-mediated apical dominance, to cytokinin supply, to strigolactone-dependent repression through BRC1, and to sugar as the initiating signal that precedes hormonal change. These are not mutually exclusive, but they imply different first targets: an intervention aimed at the wrong node in the hierarchy will be buffered by the others. In bamboo the hierarchy is entirely unresolved, which is one reason we place gibberellin-dependent internode elongation ahead of bud outgrowth control in the recommended sequence — the elongation phenotype has a single well-defined biochemical determinant, whereas bud outgrowth is a convergence point of at least four signals.

### 5.3 Quantities the literature does not yet provide

Applying the two-axis appraisal made it possible to state precisely which numbers a quantitative treatment of controllable rhizome expansion would require and which of them exist. Five are absent from the corpus reviewed here, and we set them out explicitly rather than substituting estimates.

First, annual lateral rhizome extension rate with a dispersion estimate. Expansion distances are reported for bamboo, but typically as single figures or ranges without sample size, variance, or the age class and site conditions to which they apply. Without a variance estimate, no containment target can be specified and no edited genotype can be shown to differ from a control with any stated confidence. Second, barrier breach probability as a function of embedment depth, seam treatment and inspection interval. The failure modes are well described qualitatively, but no source provides the denominators — meters of barrier, years of service, number of installations — required to convert them into a reliability function. This is the most tractable of the five gaps, since it requires the systematic compilation of existing installation records rather than new biology.

Third, absolute hormone concentrations at defined rhizome nodes. Auxin, cytokinin, strigolactone, abscisic acid and gibberellin are invoked throughout Section 2, but node-resolved absolute concentrations along an intact bamboo rhizome, distinguishing dormant, activating and elongating nodes, have not been reported. Fold-change data from mixed tissue cannot substitute, because the question is whether a gradient exists across positions at a given time, not whether a tissue changes over time. Fourth, minimum viable rhizome fragment size and its seasonal regeneration probability. Fragment-mediated re-establishment is the dominant amplification pathway for running bamboo under disturbance, yet the size threshold and the seasonal dependence of regeneration probability are not quantified for any bamboo species. This single parameter set governs both disposal protocols and the risk assessment of edited material. Fifth, the magnitude of the expansion-versus-yield trade-off. Irrigation and fertilization increase both productivity and expansion, but no study reports paired measurements across a resource gradient within a single stand, so the exchange rate between containment and production is unknown and the multi-objective index of Section 4.4 remains structurally specified but numerically unparameterized.

We regard the explicit enumeration of these five gaps as a substantive contribution rather than an admission. Each is measurable with existing methods, none requires genome editing, and together they constitute the minimum dataset on which a quantitative theory of controllable clonal expansion could be built. They are mapped onto the staged roadmap in **Table 3**.

## 6 Conclusion: unresolved questions and testable hypotheses

Rather than restate the mechanistic synthesis developed above, we close by converting it into the propositions on which it depends and by which it can be falsified. The value of a systems framework of this kind lies less in the coherence of the picture it assembles than in the precision of the predictions it licenses, and the appraisal in Section 5 makes clear that the framework is at present a well-motivated hypothesis rather than an established account of bamboo rhizome biology.

Four questions remain genuinely unresolved. Is bamboo rhizome architecture plastic within a genotype, or is the running habit canalized, so that only constitutive modification could alter it? Which signal is rate-limiting for bud outgrowth in a woody rhizome — auxin flux, cytokinin supply, strigolactone-dependent repression, or local sugar availability — given that in bamboo no hierarchy among them has been established? Can spatial restriction be achieved in a physiologically integrated clone, or does resource sharing between connected ramets necessarily propagate the consequences of a locally expressed edit? And can a transgene-free edited bamboo be produced at all in a monopodial background, given that the transformation systems that exist are concentrated in sympodial genera and that no sexual generation is available for segregating constructs away?

These questions can be sharpened into six hypotheses, each of which is testable with methods currently available and each of which would, if refuted, require a specific revision of the framework rather than its wholesale abandonment.

H1. Spacer length in bamboo is set by gibberellin-dependent intercalary meristem activity, such that local reduction of bioactive gibberellin shortens spacers without reducing the number of buds produced per unit rhizome length. Test: application of a gibberellin biosynthesis inhibitor to defined rhizome segments of intact plants, scoring internode length and node number separately. Refutation would indicate that spacer and bud-density phenotypes are not separable in bamboo and would invalidate the two-axis targeting strategy of Section 4.2.

H2. Bud outgrowth along a bamboo rhizome is gated by a local hormone gradient rather than by whole-plant hormonal status, so that dormant, activating and elongating nodes on the same rhizome differ measurably in auxin, cytokinin and abscisic acid concentration. Test: node-resolved quantification along intact rhizomes. Refutation would relocate the control point to systemic signaling and would make locally restricted editing ineffective in principle.

H3. Sugar availability, rather than hormone status, is the proximal trigger of bud release in bamboo, with hormonal change following rather than preceding it. Test: girdling or defoliation-based manipulation of assimilate supply combined with time-resolved measurement of sugar and hormone pools at the same nodes. Support would reorder the target priority toward the carbon-signaling tier despite its higher systemic risk.

H4. Transcriptional restriction of an edit to rhizome tissue does not confer physiological restriction, because clonal integration propagates altered source–sink status to connected unedited ramets. Test: carbon and nitrogen isotopic tracing between connected edited and unedited ramets, with phenotyping at increasing rhizome distance. Support would make clone-level validation mandatory for every proposal in this field and would substantially raise the evidential bar for spatially restricted strategies.

H5. Ribonucleoprotein-delivered cis-regulatory editing can produce a transgene-free bamboo with measurably reduced lateral extension and no detectable penalty in drought survival, cold tolerance, photosynthetic performance or culm mechanical properties. Test: Stage 3 of the roadmap in Section 4.5, with tissue-culture regenerants as controls. Refutation — specifically, reduced expansion accompanied by reduced stress tolerance — would indicate that expansion and resilience are not separable in bamboo and would return the problem to engineering and horticultural control.

H6. Vegetative containment endpoints dominate reproductive containment endpoints in determining the realized ecological risk of an edited bamboo, such that the minimum regenerative fragment size and the lateral extension rate predict spread better than any fertility parameter. Test: comparative monitoring of edited and unedited genets under defined disturbance regimes in confined trials. Support would justify a bamboo-specific reweighting of environmental risk assessment endpoints relative to the frameworks developed for outcrossing annual crops and regularly flowering trees.

The immediate research agenda that follows is deliberately unglamorous and does not begin with editing. It begins with measurement: a rhizome architectural census reporting spacer length, branching angle, bud density and annual extension with dispersion estimates; node-resolved hormone and sugar quantification along intact rhizomes; tissue-resolved transcriptome and small-RNA profiling from which rhizome-preferential promoters can be nominated; a retrospective compilation of barrier installation and failure records sufficient to yield a breach-probability function; and pharmacological perturbation of intact rhizomes as the first causal test in bamboo itself. Only when these are in hand does editing become a rational investment rather than a wager on orthology.

The broader claim of this review survives that qualification. Controllable rhizome diffusion should be conceptualized not as irreversible suppression, but as environmentally responsive and meristem-specific modulation that preserves clonal integration and stress resilience while constraining unwanted spread. Within this systems-based approach, bamboo and other rhizomatous plants can be repositioned from ecologically unpredictable spreaders to rationally engineered, context-adaptive landscape components. What the present appraisal adds is the recognition that this repositioning is a research program with identifiable decision points and identifiable ways of failing, not a technological inevitability, and that the most useful contribution a review can presently make is to specify exactly which measurements would move it from proposal to practice.

## Data Availability

The original contributions presented in the study are included in the article/supplementary material. Further inquiries can be directed to the corresponding authors.
